# Population and transmission dynamics model to determine WHO targets for eliminating Hepatitis C virus in Thailand

**DOI:** 10.1371/journal.pone.0309313

**Published:** 2024-10-16

**Authors:** Jennifer Astley, Sompob Saralamba, Kittiyod Poovorawan, Lisa Jane White, Ricardo Aguas, Wirichada Pan-ngum

**Affiliations:** 1 Kennedy Institute of Rheumatology, Nuffield Department of Orthopaedics, Rheumatology and Musculoskeletal Sciences, University of Oxford, Oxford, United Kingdom; 2 Mahidol-Oxford Tropical Medicine Research Unit, Faculty of Tropical Medicine, Mahidol University, Bangkok, Thailand; 3 Department of Clinical Tropical Medicine, Faculty of Tropical Medicine, Mahidol University, Bangkok, Thailand; 4 Department of Biology, University of Oxford, Oxford, United Kingdom; 5 Model Health Ltd, Oxford, United Kingdom; 6 Centre for Tropical Medicine and Global Health, Nuffield Department of Medicine, University of Oxford, Oxford, United Kingdom; 7 Department of Tropical Hygiene, Faculty of Tropical Medicine, Mahidol University, Bangkok, Thailand; Kaohsiung Medical University Hospital, TAIWAN

## Abstract

**Background:**

Hepatitis C Virus is endemic to many areas of Thailand, whose population structure is tending towards older age groups as birth rate and mortality decrease. With around 790,000 cases in 2019, prevalence is still relatively high, but the World Health Organisation has called for elimination of HCV by 2030.

**Methods:**

An age structured compartmental transmission model was used to explore the effectiveness of screening strategies with respect to WHO elimination goals, as well as the effect of changing population structure on the success or failure of such strategies.

**Results:**

Population structure did not appear to affect the timeline of elimination targets and screening individuals over the age of 30 at a high (50% per year) coverage could bring forward achievement of the incidence elimination target by four years compared to baseline (approximately 6% per year). Achievement of mortality elimination targets was not reached until after the end of the simulation (2040), and intensive screening strategies did not appear to lead to incidence elimination by 2030.

**Conclusion:**

The model suggested that with age-targeted screening programmes incidence elimination could be brought forward by several years. However, WHO elimination goals may not be met by 2030.

## Introduction

Recent studies reported approximately 760,000–790,000 Thais are seropositive for Hepatitis C Virus (HCV), half of whom are living with chronic infection [1–3]. The disease trend is declining nationally as a result of fewer new HCV infections, however, bringing patients into treatment has been slow even though the access to new curative treatment among chronic hepatitis C patients, the Direct acting antiviral (DAA), is now available and accessible to Thai population through the universal coverage scheme. Screening can be used to catch asymptomatic cases and prevent transmission, but screening coverage is low and generally only sought out once symptoms are present, many years—sometimes decades—after infection. Modelling population dynamics can provide a more detailed look at how HCV transmission relates to age and allow for more informed decisions regarding screening and treatment policies to achieve national elimination goals.

## Background

Hepatitis C is an infectious disease that primarily affects the liver, and most of the global burden exists in Low- and Middle-Income Countries (LMICs) [4]. Transmission is caused by blood contact and certain groups are at higher risk of transmitting HCV. These include individuals with HIV, men who have sex with men (MSM), injecting drug users (IDU) and prisoners [5]. In recent years, transmission has been relatively low and prevalence in southeast Asia is declining, with a national average of 0.9%. However, some previous studies reported prevalence of 7% to 15% in some specific areas and age groups in Thailand [1, 6].

A previous study reported a low HCV prevalence of 0.0%-0.90% in age groups below 30 years but one that was dramatically increasing to 3.74% in age group of 31–40 years [7]. Serious diseases such as liver fibrosis, liver cirrhosis and hepatocellular carcinoma (HCC) follow as a direct result of HCV infection [6]. These stages of liver failure can take many years to cause symptoms, and in fact only occur later in life, with the respective average ages of Hepatitis C patients with fibrosis and cirrhosis caused by HCV being 36 [8] and 52 [9]. Asymptomatically infected individuals may spend many years transmitting the disease while unaware of their status. For this reason, screening can be an effective tool for intercepting these asymptomatic cases and providing treatment to patients with earlier stages of disease, halting or slowing the transmission chain and reducing the health and economic burdens by preventing further cases.

Prior to 2019, the first-line treatments for Hepatitis C were Pegylated Interferon therapies (PEGs). The current first line treatment in Thailand is oral administration of Direct-Acting Antivirals (DAAs), which have much higher efficacy but are generally only given to symptomatic patients or those with individual screening during health check-ups and blood donation, and there is no targeted screening programme currently in place, despite suggestions that this may be necessary to reach elimination [10].

Increasing affordability for these treatments is leading researchers to believe that elimination is an attainable goal [11], and the Ministry of Public Health Thailand has called for elimination by 2030 [12]. Elimination is defined in the most recent Global Hepatitis Report as a 90% reduction in yearly incidence and a 65% reduction in yearly mortality as compared to the 2015 values [13].

The population structure of Thailand, as with many other MICs, is changing as mortality amongst older age groups decreases rapidly [14], and as such the proportions of older age groups are growing while younger groups diminish. HCV disproportionately affects older age groups [6], hence it may be useful to consider these heterogeneities within a population when considering the disease’s impact and possible intervention and treatment options.

Previous HCV transmission models in Thailand have not considered age or population dynamics, rather modelling the population homogeneously, assuming all individuals are equally susceptible to infection and different disease stages [15, 16]. The 2016 model also assumed logistic population growth in Thailand, which is inconsistent with recent population projections [17, 18].

The model in this report incorporates changing population structure and dynamic birth and death rates into an HCV transmission model to explore national-level screening policies. Aspects of this modelling could be used to study other disease transmission dynamics in Thailand, and with sufficient data could be applied more broadly to different population projections. Furthermore, with data on other population dynamics on specific groups relevant to disease transmission, this approach could be used to model the targeting of risk groups as well as age groups. Since 2023, the national policy in Thailand has started implementing the screening programme [19], in which high-risk individuals can be screened once a year and those born before 1992 can be screened once in a lifetime, however due to the novelty of this policy, few data on these groups exist. Additionally, the future of the population structure of Thailand continues to change and there is considerable uncertainty in how it will look in the immediate and near future.

This study employs a novel, age-structured HCV transmission model to investigate the effect of changing population structure on the effectiveness of baseline and targeted screening programmes and explore whether elimination goals outlined by the World Health Organisation are feasible in the timescale suggested. The advantage of this model is its capacity to incorporate data as it becomes available and to monitor the effect of different screening schemes to inform national-level policy.

## Methods

### Transmission model

An age-structured compartmental model was used to model progression through the transmission cycle that accounted for population dynamics, with screening and treatment programmes represented in the model. The HCV transmission portion was adapted from the work of Poovorawan et al., 2016 and the age structure from an otherwise unrelated disease transmission model [20].

**Fig 1:**
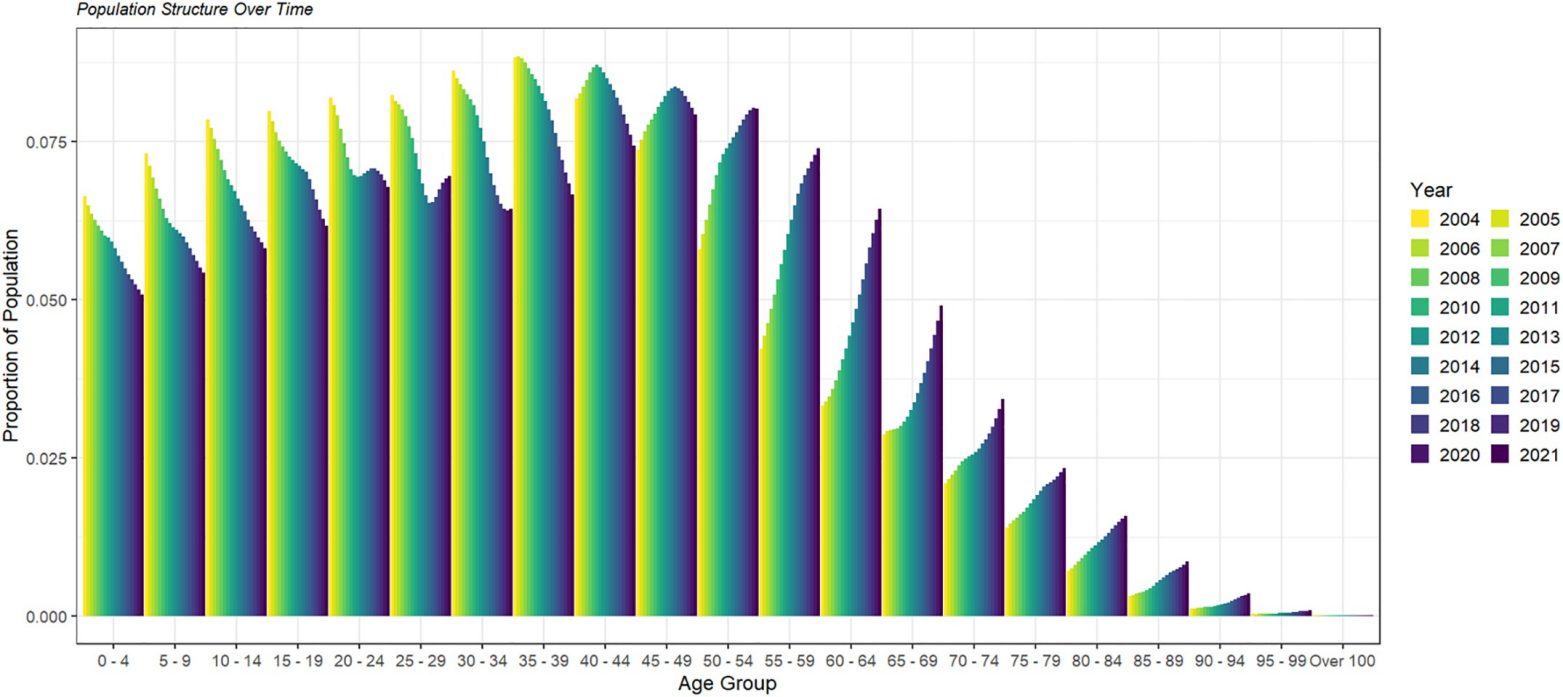
Compartment diagram showing the HCV transmission cycle modelled as well as the embedded age structure. Individuals travel through both simultaneously. 21 5-year age groups were modelled, from 0–4 to 100+.

Fig 1 shows the general structure of the transmission and age compartments of the model. A full list of equations, compartments and parameters can be found in S2 File, with the full model code available at https://github.com/astleyjennie/HCVinThailand. The system of equations in S2 File was implemented in R and solved using the initial conditions given in S1 File. The Runge-Kutta 4^th^ order method was used with the General Solver for Ordinary Differential Equations in the deSolve package. An internal time step of h = 0.1 was used to minimise divergence and allow efficient solution of the system.

### Age structure

Each of the compartments representing the various stages of HCV transmission and treatment from a previous model by Poovorawan et al., 2016 were stratified into 21 age groups: 0–4, 5–9, …, 95–99, 100+. A 21x21 matrix was initialized representing travel through the aging compartments: travel was only possible from the previous age group and into the next one. An individual moved through the age groups at a set aging rate (0.2 per year i.e. spending 5 years in each 5-year age compartment) while simultaneously moving through the transmission cycle at rates determined by transition parameters from the previous model (defined in S2 File). This was implemented by initialising empty vectors of length 21 for each of the disease compartments and then solving the ODEs with each compartment vector. The flow of new births was injected into the first entry of the susceptible compartment, representing newborn babies entirely susceptible to HCV infection. Individuals moved out of the age group due to multiplication of each compartment by the aging matrix and died of natural causes due to the multiplication of each compartment by the natural death matrix.

### Population demographic data

**Fig 2:**
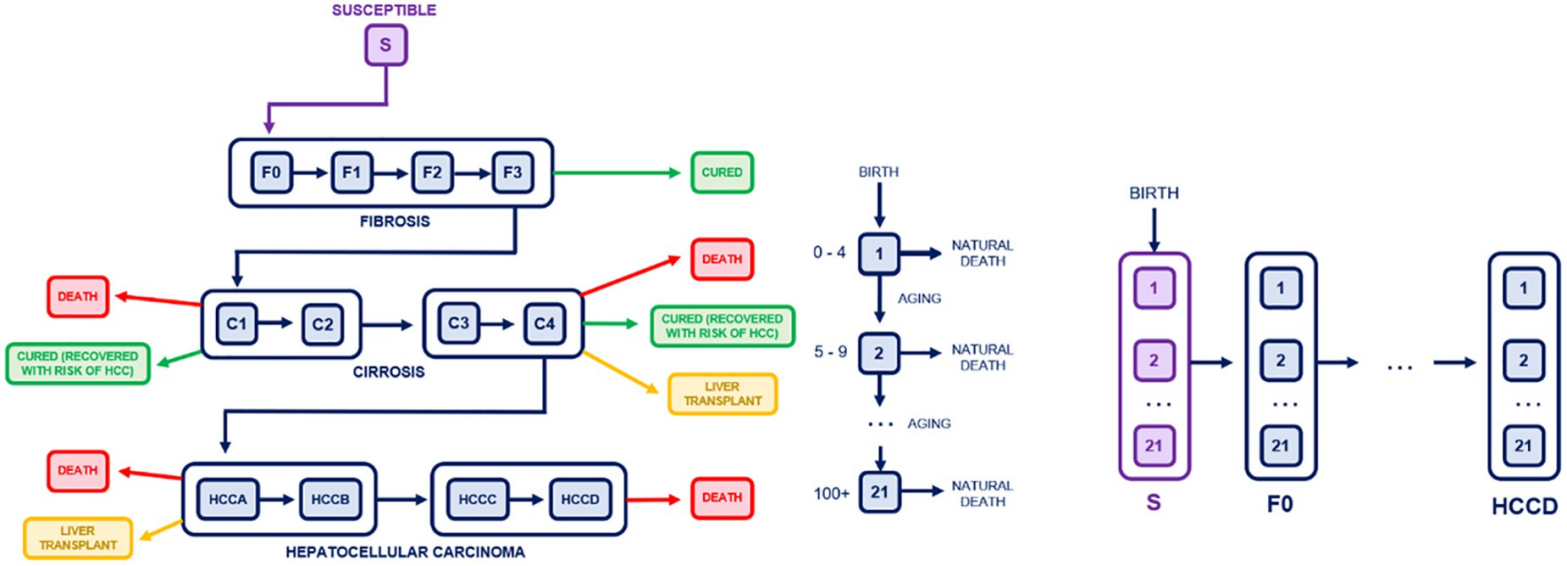
Population structure of Thailand by age group [17]. Note the clear aging of the population: age groups below 44 are declining while those above 50 are growing.

United Nations demographic data were used for the years 2004 to 2021 to visualise the population dynamics of Thailand and to calibrate the model. Population structure, birth and death rates (per person per year) were recorded, and UN projections were considered [17]. Fig 2 shows the proportion of each age group in Thailand. S1 Fig shows total population and birth rate data and projections [18].

### Natural mortality and birthrate

We calculated mortality rates from population data and used these values for the years 2004–2021. The multiplier for each year (mortality in 2005 divided by mortality in 2004 etc.) and the mean was calculated for each age group to obtain the average factor by which each age group’s mortality had increased (or decreased) between 2004 and 2021. These values were multiplied by a factor (0.975) to fit the overall population data projections, implying that mortality rates will continue to decrease by 2% and 3% per year for individuals aged 0–49 and 50+ compared to the 2004–2021 average. The data available for births per person per year were given a multiplication factor in order to ensure the fit to overall population data.

### Model calibration: Population demographics

We chose multiplication factors for mortality rates and birthrates in order to generate four population scenarios (detailed in S1 File), allowing for uncertainty within the available projections. These four population scenarios were compared to investigate the effect of population structure on HCV transmission, elimination and screening strategy success. The mean historical natural death rates (deaths per person per year) from 2004–2021 were further decreased by 2% per year for ages 0–49 and 3% per year for ages 50+ from 2022 onwards in order to be consistent with Thailand’s aging population trend, while historic and projected birth rate values (births per person per year) were multiplied by a single value in order to approximate the overall projected trend in the Thai population [17, 18]. This was taken as baseline and used for the main result. Three other modifications on age-specific mortality rates and birth rates were explored. More details about the population scenarios can be found in S2 File.

**Fig 3:**
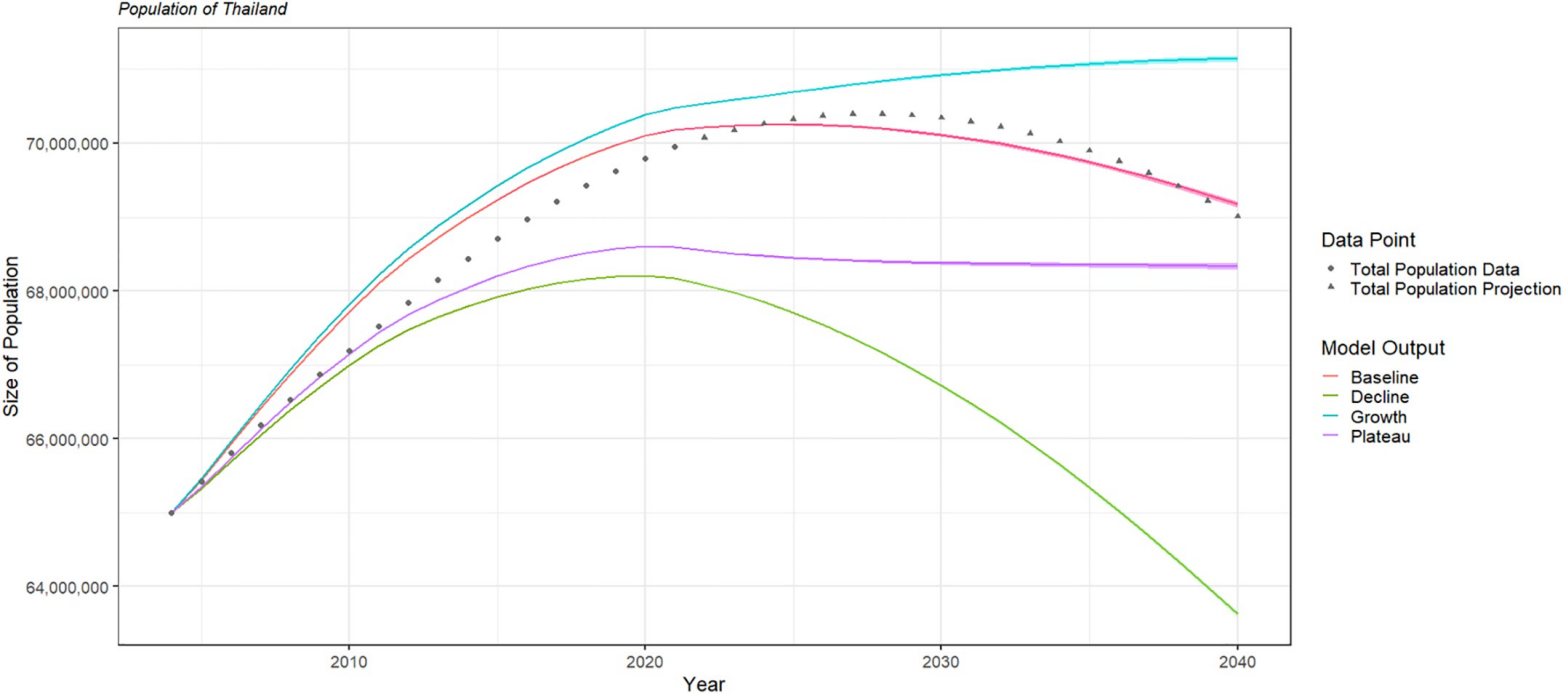
The model output of the total population of Thailand for each of the four population structure scenarios. The narrow ribbons show the 95% confidence interval based on the uncertainty in baseline coverage. The points show the United Nations data and projection for Thailand’s population.

**Fig 4:**
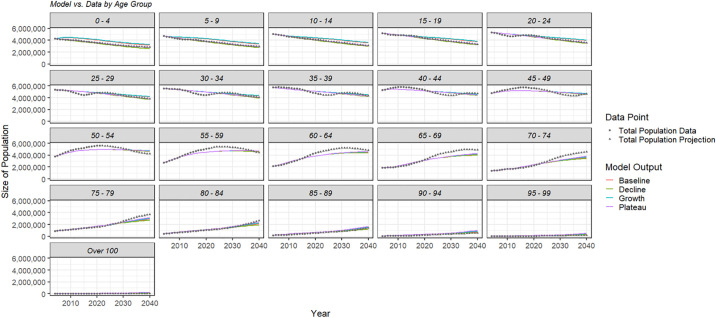
The model output of population by age group for each of the four population scenarios overlaid with United Nations data and projections.

Figs 3 and 4 show the model output for total and age-group populations with historic and projected values. The baseline scenario does not adequately capture the data; however, sensitivity analyses were performed to investigate the effect of population trends on other epidemiological quantities of interest and did not significantly affect any results. These results can be found in S3 File.

### Transmission and contact matrix

The contact matrix was derived from data on sexual partners and sexual contact between age groups from a study on Human papillomavirus infection (HPV) in Laos [21]. Although more complete contact matrices exist describing other types of contact between age groups [22], very limited data are available on the particular type of contact that transmits HCV; namely sexual and blood contact. Although not specific to HCV and Thailand, the contact matrix for a sexually transmitted disease in a Southeast Asian setting was deemed the most appropriate for the purposes of this model, given the increasing trends in unsafe sex with multiple partners and chemsex activities among MSM reported in Asia [23, 24]. S2 Fig shows a heat map of the transmission matrix *beta*.

### WHO elimination targets

Prevalence (%) was defined as 100 times the total number of cases (F, C and HCC compartments) over total individuals in that age group or population, while the incidence was defined as the total number of new cases of HCV in the given year. The elimination targets outlined in the Global Hepatitis Report [13]–a 90% reduction in new cases and a 65% reduction in HCV related death compared to the 2015 baseline—refer only to global values, and no Thailand-specific values were given for the 2015 baseline. The 2015 values of the model were used to calculate 2030 incidence and mortality elimination targets due to a lack of data.

### Model calibration: Epidemiological data

Epidemiological data on HCV in Thailand are very scarce, especially by age. Sources report both the prevalence active HCV infection and of anti-HCV antibodies, a proportion of which represent active infection in the Thai population [5, 25]. Throughout this investigation, data on anti-HCV infections were used for prevalence calculations, in line with previous HCV transmission modelling work [16]. This study reports the age distribution of HCV cases in a high prevalence area of Thailand and extrapolates whole-country values, providing a relatively reliable distribution of infection across age groups. The work of [2] was used to calibrate the model as the study includes relatively complete national prevalence data stratified by age groups for 2004 and 2014. The 2004 values were used to initialize the model and the 2014 values were used for model calibration. The exact screening coverage from 2004–2021 is unknown, so a model fit was performed using the limited prevalence data available. A Negative Log Likelihood function was used with *optim* to estimate the value of the baseline screening coverage between 2004 and 2022 that best fit the age-stratified prevalence data in 2014 at the baseline population scenario. The baseline screening coverage was assumed to be uniform across age groups, and the result of the fit was a baseline screening coverage of 6.18% ± 1.22% per year. Although coverage is not expected to be uniform, insufficient data are available to assume otherwise, and this distribution could be modified if more information on screening coverage was known. The 95% confidence intervals relating to all further calculated epidemiological quantities (prevalence, incidence, mortality) were calculated using the uncertainty in the baseline screening coverage.

**Fig 5:**
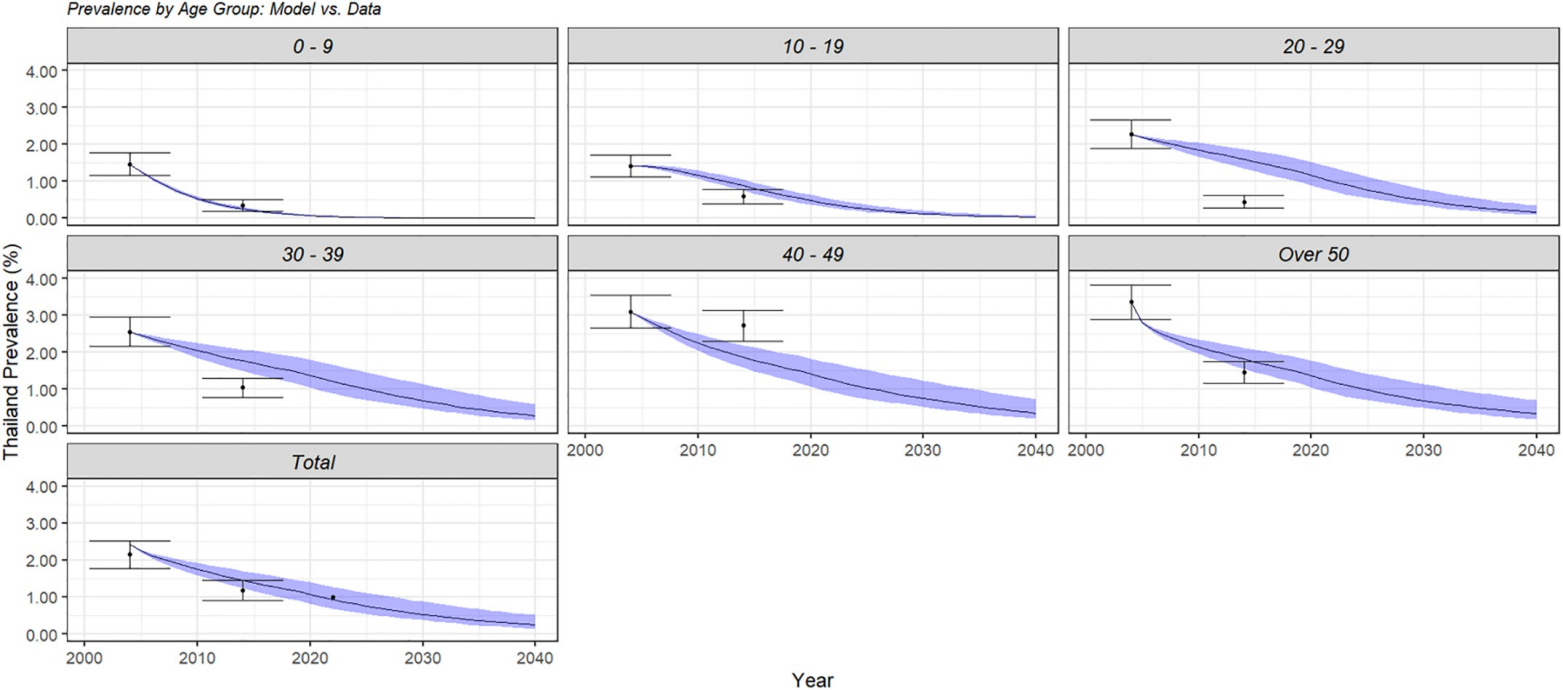
Model output compared with 2004 and 2014 prevalence data by age group at baseline population. Data error bars were calculated using sample and population size from the 2004 data. The 95% confidence interval ribbons were based on uncertainty in baseline screening coverage. Note the total prevalence in 2004 is slightly higher due to differences in total population value between the data source used for prevalence and population. Also note the difference in fit across age groups due to the assumption that baseline screening is uniform. 2022 prevalence data was available for the total population only.

The model output was adapted to produce prevalence by the same aggregated age groups given in the 2004 and 2014 data, with 10-year age groups up to 49, and all other cases grouped into 50+. Each prevalence value was calculated by summing the infections over all relevant age groups and dividing by the total number of individuals in those age groups in the given year at baseline screening and population. These data and calculations can be found in S1 File, with more information in S2 File. The model showed a reasonably good reproducibility level of two available points of prevalence data, as shown in Fig 5.

### Treatment

We used the current first-line treatment (DAAs) from 2019 onwards in the model, and the previous standard treatment (PEGs) from 2004 (the beginning of the model) to 2019. The target population for the treatment intervention was any individual in the liver fibrosis (F0-F3) and cirrhosis (C1 for PEGs, C1-C4 for DAAs) compartments i.e., anyone with active HCV infection that had not yet progressed to HCC, in line with the original HCV model. Parameters relating to HCV transmission and treatment from the work of [16] were used.

### Screening strategies

Four target age groups at four yearly screening coverages including the current baseline (6.18% across all age groups) were compared to investigate whether the national elimination goals appeared to be achievable in the desired time frame. Low (15%), medium (25%) and high (50%) values were chosen for yearly coverage, targeting individuals aged 30+, 40+, 50+ and 60+, starting in 2023 at a duration of 7 years to reflect the 2030 elimination target. Age groups not actively targeted in a given strategy were screened at baseline. Individuals in F and C compartments are treated according to test sensitivity parameter value. We assumed uniform screening coverage across a given age or disease stage compartment.

### Model assumptions

Inherent in the use of a compartmental transmission model is the assumption that all individuals in a single compartment are identical. Stratification of the previous model into age groups has mitigated this assumption somewhat, but there are still limitations to the conclusions that can be drawn from such a model. The model assumes spatial homogeneity across Thailand, whereas prevalence is in fact concentrated in certain endemic regions [6]. The limited data available were used to initialise the model in the year 2004, and assumptions were made about younger age groups being constrained to the earlier stages of disease (see S2 File). Little to no reliable data was available on the distribution of age groups across the stages of fibrosis, cirrhosis and HCC, so an early initialisation (2004) compared to the present day (2022), along with calibration to 2014 data was imposed to mitigate this assumption: transition rates between liver stages over a period of 18 years was assumed to stabilise results enough to make the necessary conclusions about 2023 onwards, however further data on this distribution could improve the accuracy of the model.

There are some data available on spatial heterogeneity of HCV prevalence in Thailand, as well as the age distribution within higher prevalence areas of the country. The objective of this model was to inform national level policy and as such we have not included any spatial elements to our model. The model could however be adapted to specific regions of Thailand and used to explore specific area policy.

The model assumed that an individual in a disease compartment will be treated if they are targeted by the screening programme coverage, and that all individuals requiring treatment will receive it. Due to Thailand’s universal healthcare system this assumption is reasonable, however it is noted that not all those who need treatment receive it.

### Technology

R Studio^®^ version 2021.09.0 Build 351 was used to run the model. R packages used were: pacman, tictoc, Hmisc, viridis, deSolve, tidyverse, doParallel, manipulate, readxl, gridExtra, grid, scales. Microsoft^®^ Excel^®^ 2019 MSO (16.0.10387.20023) 64-bit was used to store and manipulate data, initial conditions, results and scenarios.

More details about the methods involved in the model setup as well as all code for running and fitting the model can be found in supplementary materials.

## Results

The success of each screening strategy with respect to elimination target years did not vary significantly between population growth scenarios, in both HCV incidence and HCV-related death. In all four population scenarios, the current baseline screening strategy with the mean estimate of coverage (6.18% per year across all age groups) did not reach incidence elimination until 2039 or beyond the simulation (after 2040). The mortality elimination goal was not reached until after the end of the simulation for all screening strategies and population scenarios. At the baseline screening strategy, the total number of deaths increased in the plateau and growth population scenarios, with 541 and 231 more deaths than the baseline population.

At baseline population scenario, increasing coverage from 15% to 50% per year for individuals age 30+ brought forward the year of incidence elimination by 4, from 2038 to 2034, while incidence elimination was not reached until beyond the simulation for 50+ and 60+ groups at all coverages. The difference in incidence elimination years between baseline and the most extreme screening strategy (individuals age 30+ at 50% per year) was at least 6 years (>2040 to 2034). This strategy led to the highest number of cases and deaths averted (11,049 cases and 10,558 deaths averted). This involved the highest level of excess screening, at 300,790 individuals over the 7-year period.

**Fig 6:**
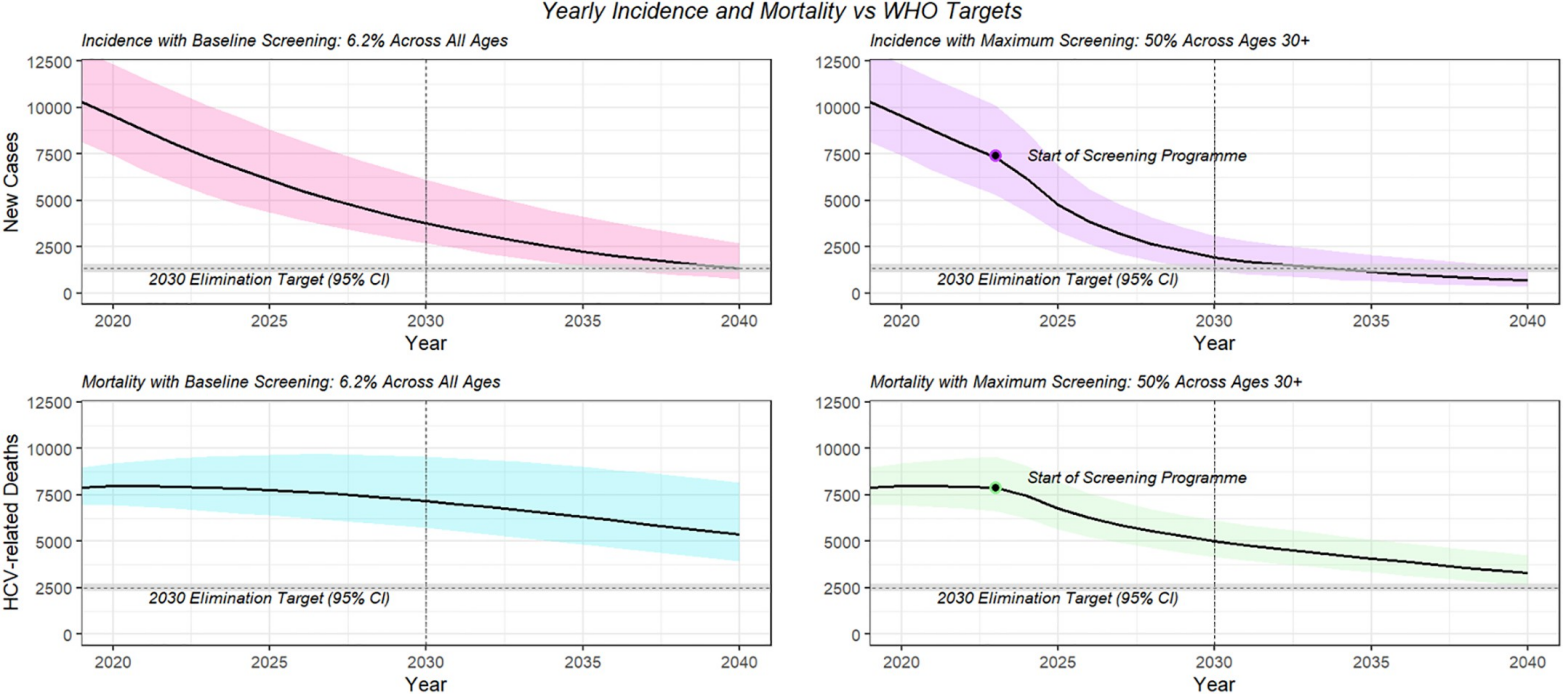
Model output for incidence and deaths compared with the WHO elimination goals for baseline and maximum screening programmes. The ribbons show 95% interval as given by the uncertainty in the baseline screening coverage.

Yearly incidence (total new cases per year) and mortality (HCV-related deaths per year) for the baseline and most extreme screening strategies are shown in Fig 6. The results of the baseline population strategy are shown in Table 1. Full results of all scenarios and strategies can be found in S3 File.

**Table 1:** Results of all screening strategies at baseline population scenario including 95% CI based on uncertainty in baseline screening coverage.

Screening Strategy	2030 Incidence Target Estimate	Incidence in 2030	2030 Deaths Target Estimate	Deaths in 2030	Total Cases	Total Deaths	Total Screened	Year Incidence Elimination Target Reached (Mean)	Earliest Year Incidence Elimination Target Reached (Lower 95% CI)	Latest Year Incidence Elimination Target Reached (Upper 95% CI)	Year Mortality Elimination Target Reached
**Baseline**	**1333**	3764	2510	7166	35882	52791	188188	**> 2040**	**2038**	**> 2040**	**> 2040**
30+ at 15%	1333	2989	2510	6265	32117	49111	322730	**2038**	**2035**	**> 2040**	**> 2040**
30+ at 25%	1333	2473	2510	5661	29113	46226	407930	**2036**	**2033**	**> 2040**	**> 2040**
30+ at 50%	1333	1939	2510	5027	24833	42233	488978	**2034**	**2031**	**2039**	**> 2040**
40+ at 15%	1333	3371	2510	6383	33964	49532	291935	**> 2040**	**2036**	**> 2040**	**> 2040**
40+ at 25%	1333	3104	2510	5859	32416	46973	359018	**2039**	**2035**	**> 2040**	**> 2040**
40+ at 50%	1333	2812	2510	5308	30168	43420	425915	**2038**	**2034**	**> 2040**	**> 2040**
50+ at 15%	1333	3608	2510	6557	35116	50209	257760	**> 2040**	**2036**	**> 2040**	**> 2040**
50+ at 25%	1333	3500	2510	6147	34493	48173	303858	**> 2040**	**2036**	**> 2040**	**> 2040**
50+ at 50%	1333	3379	2510	5713	33578	45328	352426	**> 2040**	**2036**	**> 2040**	**> 2040**
60+ at 15%	1333	3716	2510	6775	35643	51117	222742	**> 2040**	**2036**	**> 2040**	**> 2040**
60+ at 25%	1333	3682	2510	6509	35445	49786	243976	**> 2040**	**2036**	**> 2040**	**> 2040**
60+ at 50%	1333	3642	2510	6219	35148	47900	262190	**> 2040**	**2036**	**> 2040**	**> 2040**

## Discussion

The results showed that the four population structure projections modelled do not appear to significantly affect the elimination year of any of the screening strategies modelled: a screening strategy reaches incidence elimination target values at roughly the same time regardless of changing population demographics, and epidemiological quantities of interest (incidence, mortality and prevalence in 2030) also remain largely unchanged between scenarios (see S3–S5 Figs). This is likely because the strategies were based on a percentage coverage, so as the number of older individuals increased, so did the amount of excess screening. However, the aging population scenarios (growth and plateau) lead to a higher number of deaths and cases overall, due to HCV disproportionately affecting older individuals. Suppose the population projection of Thailand changes due to fertility campaigns or further decreasing mortality, or indeed in other countries where population structure is volatile and changing rapidly. In that case, consideration of population dynamics and structure may not be necessary when proposing screening programmes and policies. However, more HCV-related deaths and cases could be expected in an aging population.

The model showed that yearly screening coverage might impact elimination results less than the targeted age group; in fact, even radical screening strategies may only bring incidence elimination forward by a few years. The most effective group to target appeared to be individuals aged 30+ (the largest group targeted), but even at a high coverage of 50% per year this strategy did not achieve the WHO 2030 target in the model. Note that the model underestimated the prevalence of HCV in the 20–29- and 40–49-year age group due to the assumption that baseline coverage was uniformly distributed across all ages, so in reality the most effective age group to target may in fact be different. Additional age-stratified epidemiological data could substantiate this. S6 and S7 Figs show incidence and mortality results for all screening strategies at the baseline population scenario.

The screening strategies modelled would undoubtedly require a great deal of resources to achieve such coverage of a large population due to the high cost of screening, which may not be feasible within government budgets and resource allocation. The screening strategies with the highest success (cases and deaths averted, year of incidence elimination) require a large amount of excess screening. Further in-depth economic evaluation would be required to investigate the impact of such screening strategies compared to the current baseline.

The work in this study could also be built upon by applying the age-stratified transmission structure and changing population demographics to other populations. Better fitting of the model could be performed if more data become available. The model could be modified to focus on risk groups that are disproportionately affected by HCV as well as older age groups, such as MSM (Men who have Sex with Men), prisoners and IDU (Injecting Drug Users), with the availability of data on age and prevalence distribution of and transmission-related contact between these groups. We note that the choice of a sexual contact matrix to inform transmission may not best reflect the type of contact known to spread HCV, namely needle sharing and iatrogenetic processes. However, higher-risk sexual contact is known to contribute to some transmission and as such, due to a lack of more appropriate options, the sexual contact matrix was chosen over a matrix describing a less clinically relevant form of contact e.g. face-to-face.

Data surrounding age stratification of HCV cases and deaths in Thailand are significantly lacking, leading to a large amount of uncertainty in the results, and this limitation is recognised. Mortality decreased around 2020 both in data and in another HCV-related deaths model [15] (see S8 Fig) as expected due to the new treatment programme implemented in 2019. However, the model in this report underestimated the number of deaths caused by HCV compared to the 2019 model, and mortality elimination targets were still not met in the timescale simulated. This underestimation is likely due to the assumption that all individuals requiring treatment receive it, when many do not receive treatment until the fatal stages of liver failure, by which point treatment is ineffective. More robust conclusions can be drawn about the efficacy of screening programmes on mortality based on the results of this study. However, it appears that Thailand’s WHO mortality goal may still need to be reached by 2030.

This model suggested that the elimination of HCV (defined by WHO as a 90% reduction in new cases and a 65% reduction in HCV-related death from the 2015 baseline) can be achieved in Thailand, and the introduction of screening programmes could be brought forward by several years. However, none of the simulations run here resulted in the elimination year of 2030 or earlier. This result held for a variety of realistic population projections, and as such the consideration of variations in population structure may not be high priority when making screening policy decisions.

## Supporting information

S1 Fig Birth rate and population. Figure showing birth rate and population data and projection from United Nations data.
(PNG)

S2 Fig Beta matrix: Derived from sexual contact and HPV in Laos. Heat map of the beta transmission matrix derived from sexual contact data from a study on HPV in Laos.
(PNG)

S3 Fig Sensitivity analysis: Effect of population scenario on 2030 incidence. Bar chart showing the difference in 2030 incidence for each population scenario, screening coverage and targeted age group.
(PNG)

S4 Fig Sensitivity analysis: Effect of population scenario on 2030 mortality. Bar chart showing the difference in 2030 mortality for each population scenario, screening coverage and targeted age group.
(PNG)

S5 Fig Sensitivity analysis: Effect of population scenario on 2030 prevalence. Bar chart showing the difference in 2030 prevalence across all age groups for baseline and most extreme screening strategies between population scenarios.
(PNG)

S6 Fig Yearly HCV incidence by target age group and screening coverage. Model output of incidence compared to WHO 2030 goals for all screening coverages and target age groups at baseline population scenario.
(PNG)

S7 Fig Yearly HCV-related mortality by target age group and screening coverage. Model output of mortality compared to WHO 2030 goals for all screening coverages and target age groups at baseline population scenario.
(PNG)

S8 Fig Yearly HCV-related mortality by population scenario. Model output of mortality at baseline screening coverage for all four populations scenarios compared with 2030 WHO goals and model output from [13, 15].
(PNG)

S1 File HCV model data. Excel file containing all data (raw and manipulated), scenarios, simulations and results.
(XLSX)

S2 File Supplementary methods. Complete list of all Ordinary Differential Equations specifying the transmission model. Complete list of all compartments in the transmission model. Complete list of all parameters in model with description, parameter name (in model code), value and source. Further detail on methods used in all aspects of the work done to produce these results.
(DOCX)

S3 File Supplementary results. Further detail on results obtained from this model that are additional to main results.
(DOCX)

S1 Graphical abstract (TIFF)
