# Supplementary figures and images for: Population and transmission dynamics model to determine WHO targets for eliminating Hepatitis C virus in Thailand
Source: PLoS One. 2024 Oct 16;19(10):e0309313. doi: 10.1371/journal.pone.0309313 (PMC11482681; doi:10.1371/journal.pone.0309313)

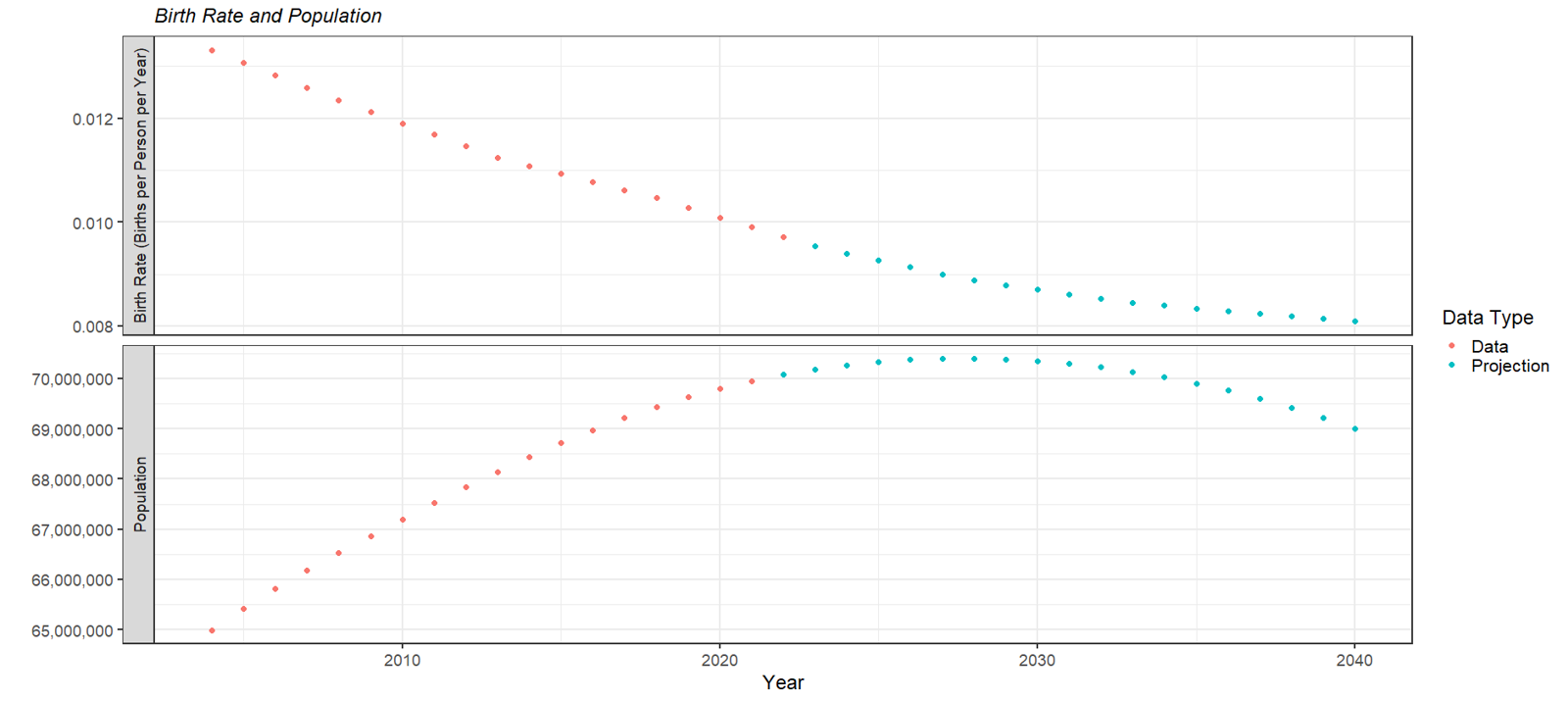

Supplement: S1 Fig — Figure showing birth rate and population data and projection from United Nations data. (PNG) [file pone.0309313.s001.png]

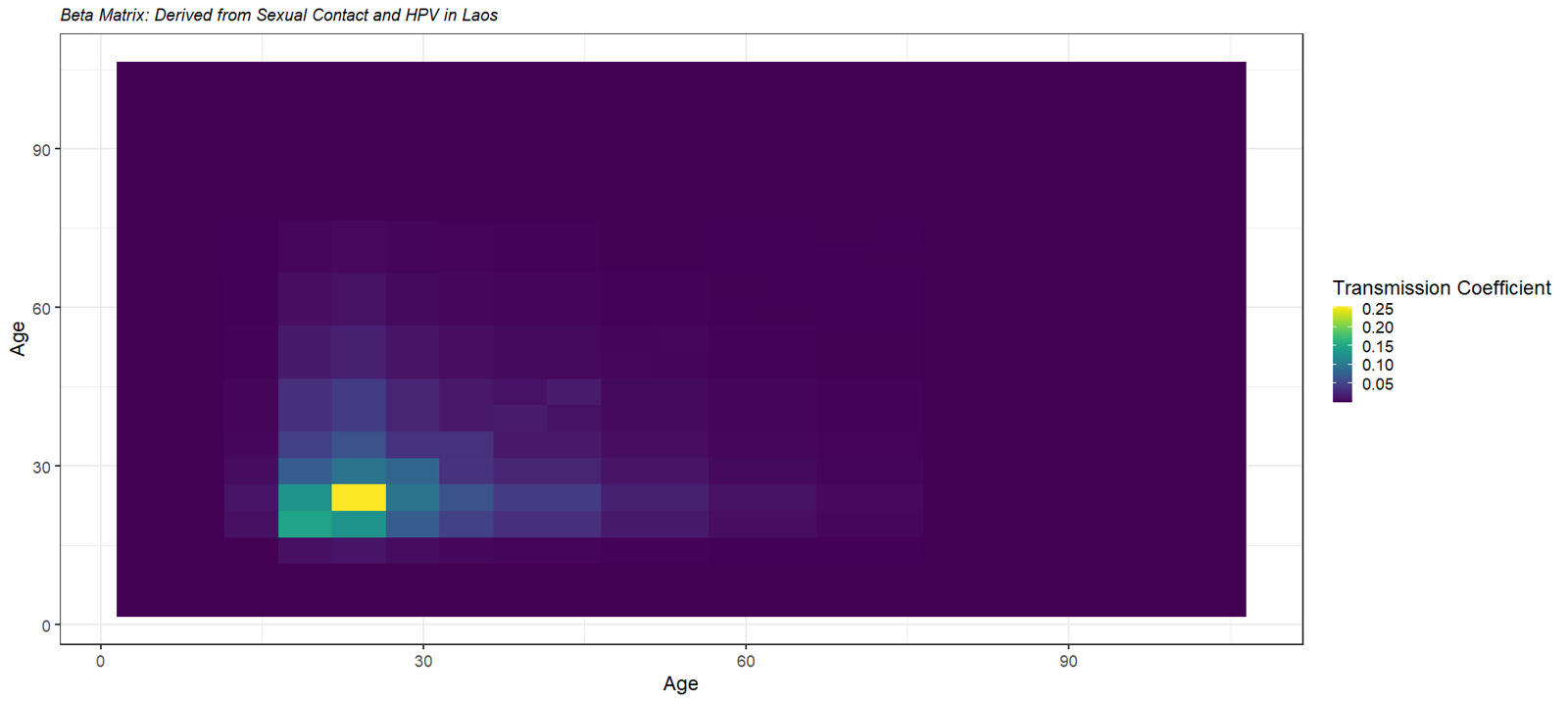

Supplement: S2 Fig — Heat map of the beta transmission matrix derived from sexual contact data from a study on HPV in Laos. (PNG) [file pone.0309313.s002.png]

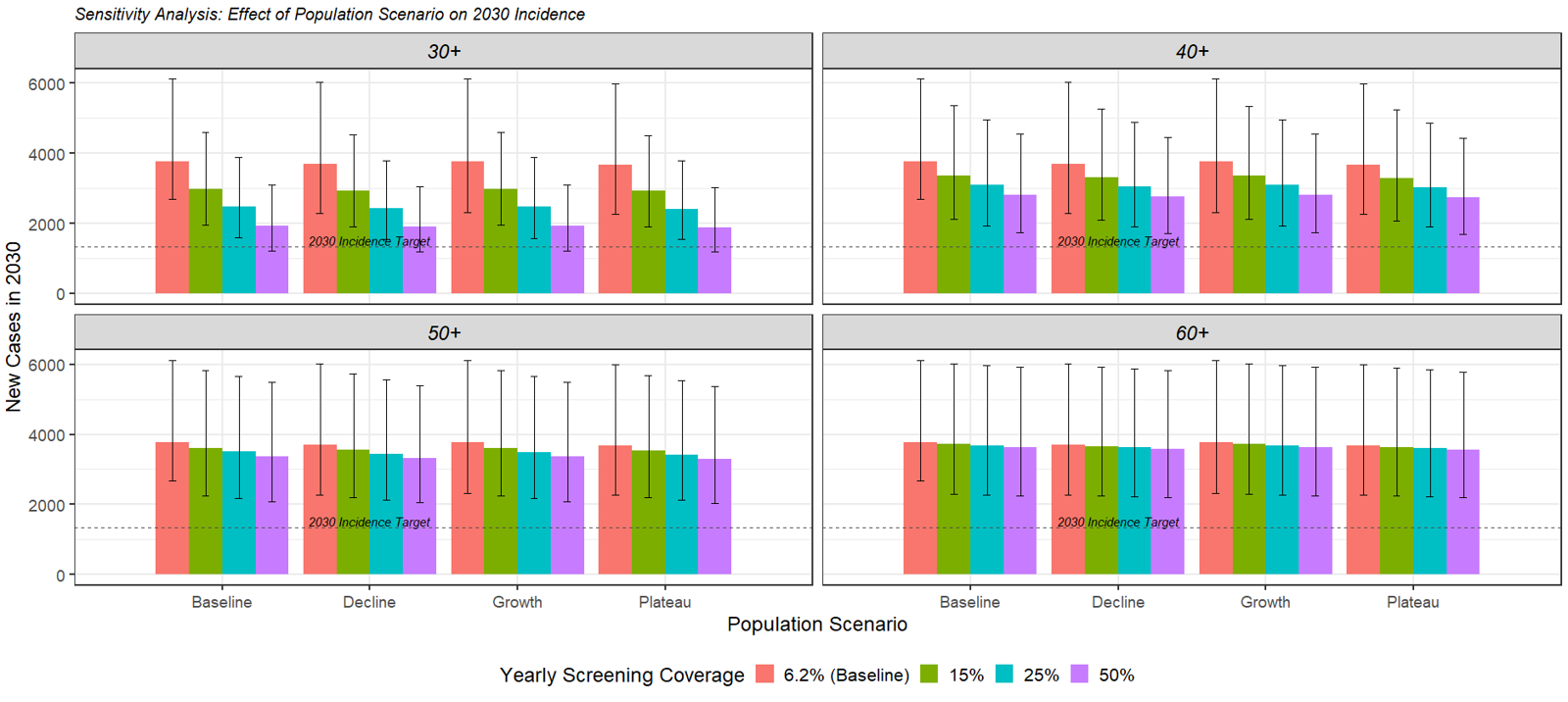

Supplement: S3 Fig — Bar chart showing the difference in 2030 incidence for each population scenario, screening coverage and targeted age group. (PNG) [file pone.0309313.s003.png]

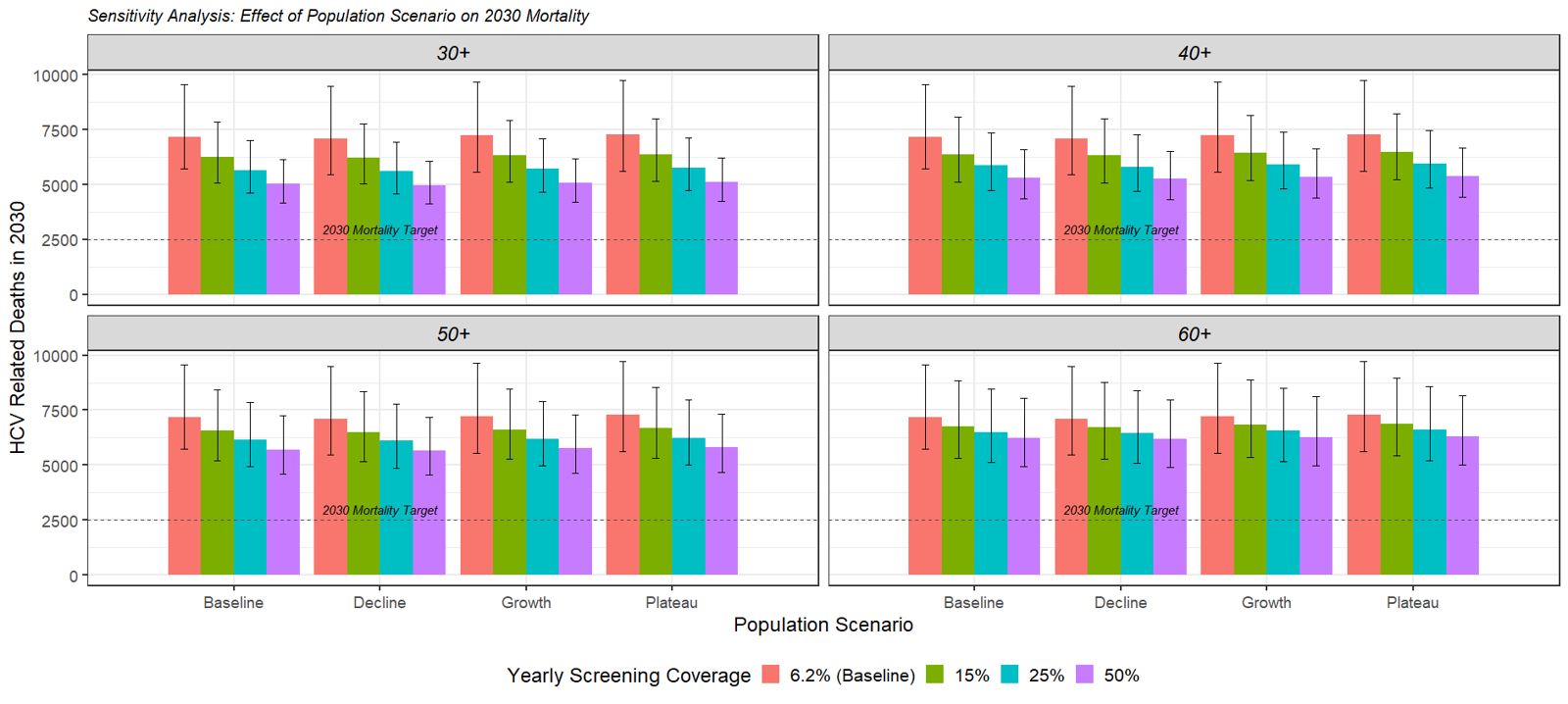

Supplement: S4 Fig — Bar chart showing the difference in 2030 mortality for each population scenario, screening coverage and targeted age group. (PNG) [file pone.0309313.s004.png]

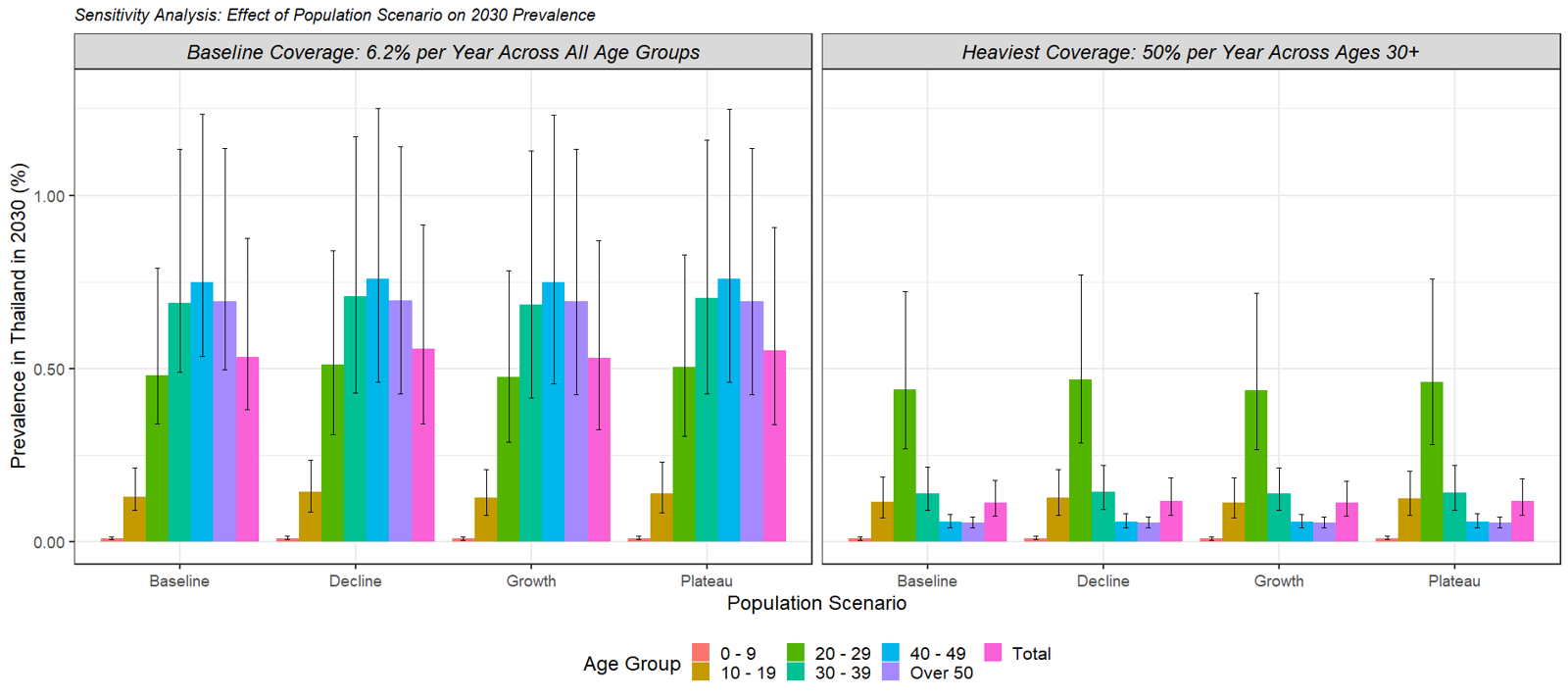

Supplement: S5 Fig — Bar chart showing the difference in 2030 prevalence across all age groups for baseline and most extreme screening strategies between population scenarios. (PNG) [file pone.0309313.s005.png]

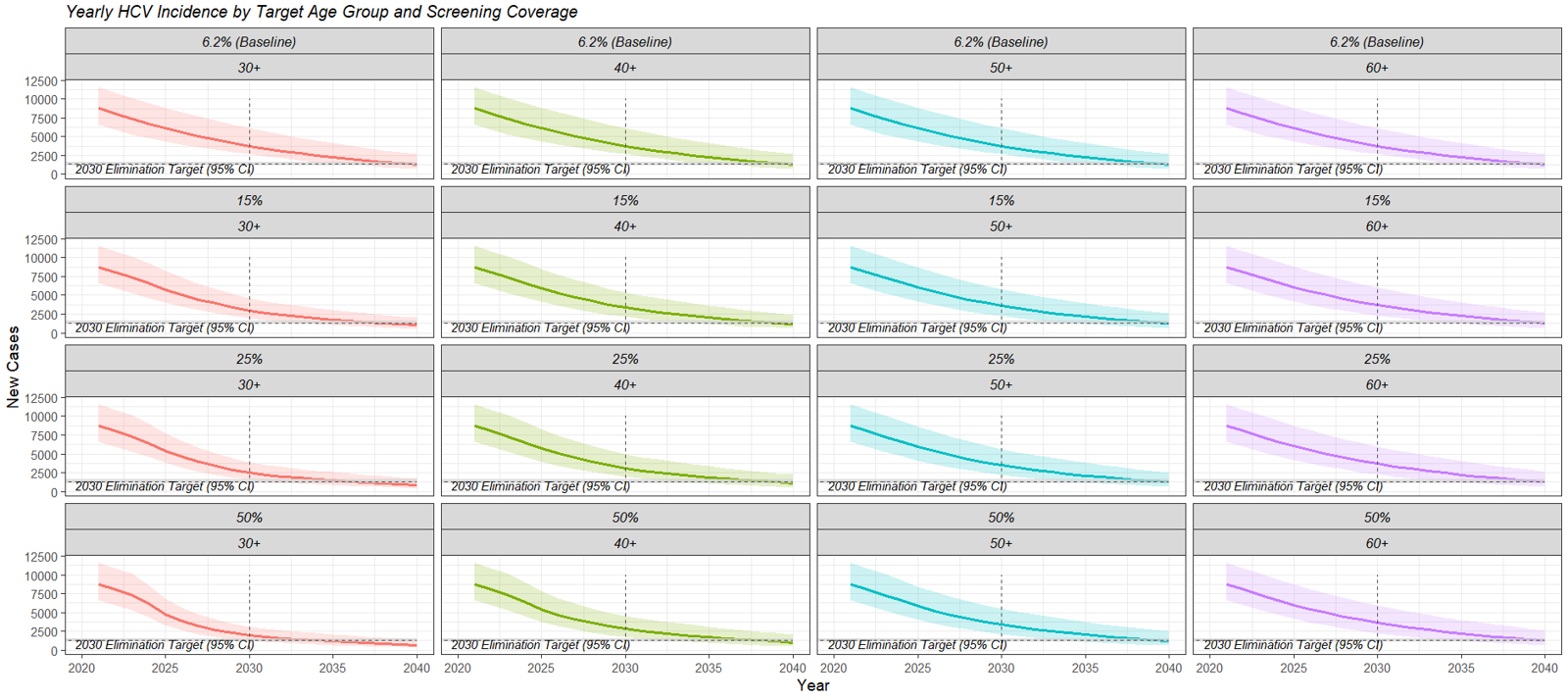

Supplement: S6 Fig — Model output of incidence compared to WHO 2030 goals for all screening coverages and target age groups at baseline population scenario. (PNG) [file pone.0309313.s006.png]

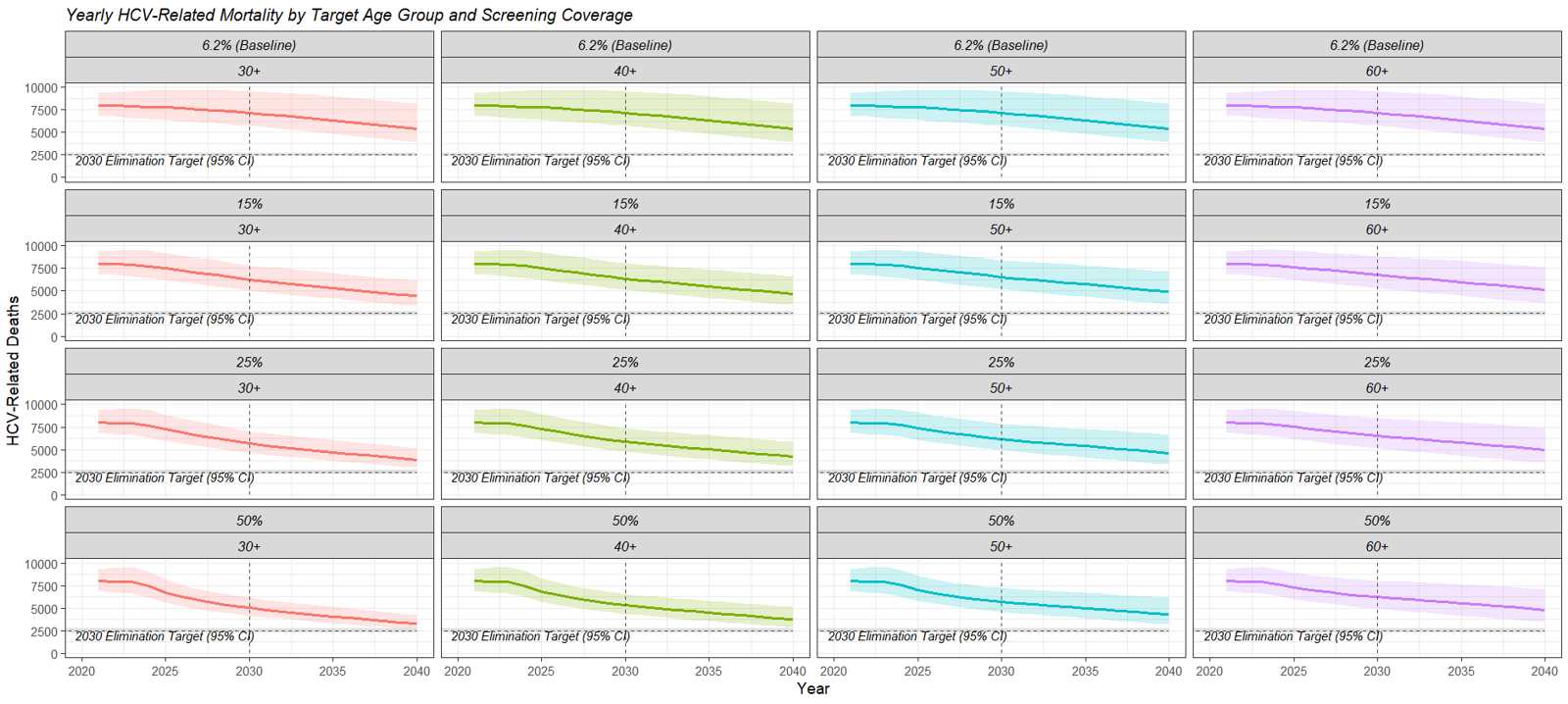

Supplement: S7 Fig — Model output of mortality compared to WHO 2030 goals for all screening coverages and target age groups at baseline population scenario. (PNG) [file pone.0309313.s007.png]

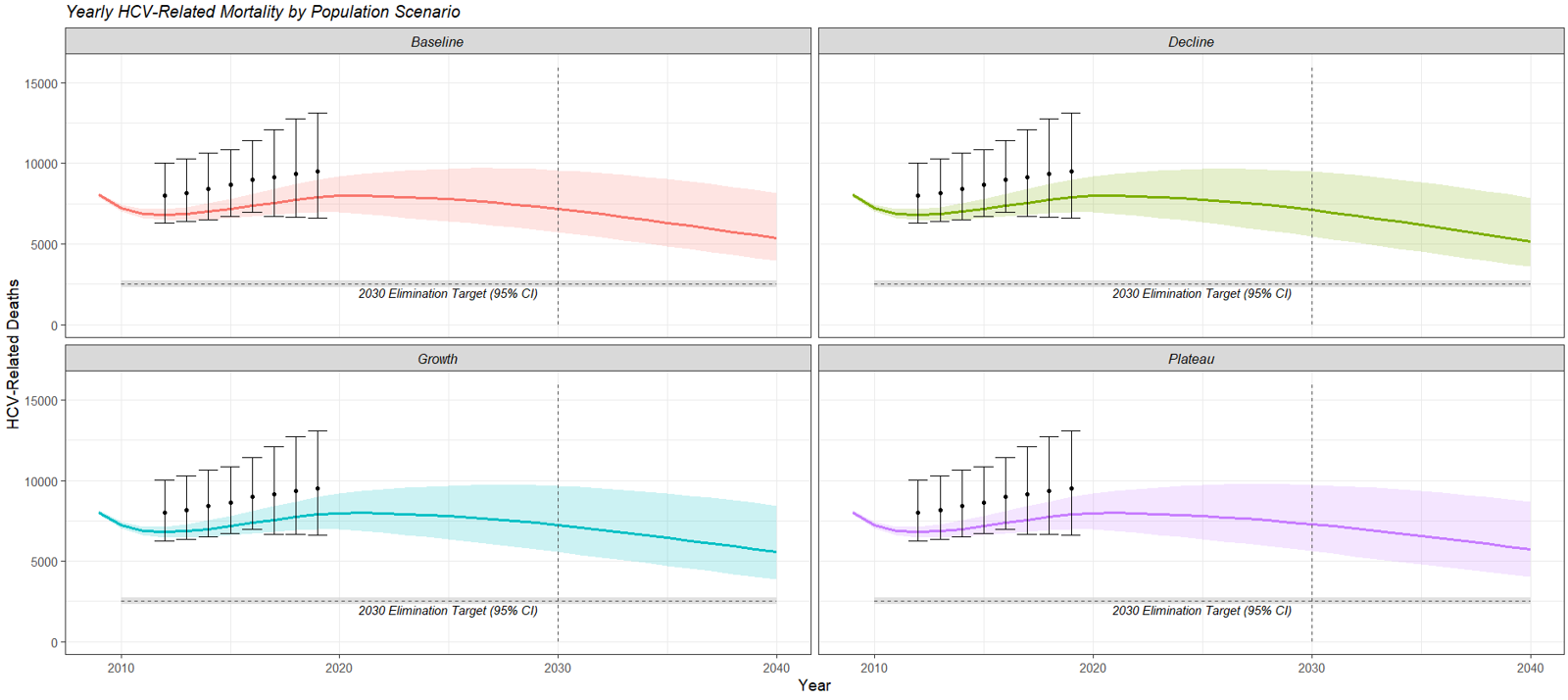

Supplement: S8 Fig — Model output of mortality at baseline screening coverage for all four populations scenarios compared with 2030 WHO goals and model output from [13, 15]. (PNG) [file pone.0309313.s008.png]

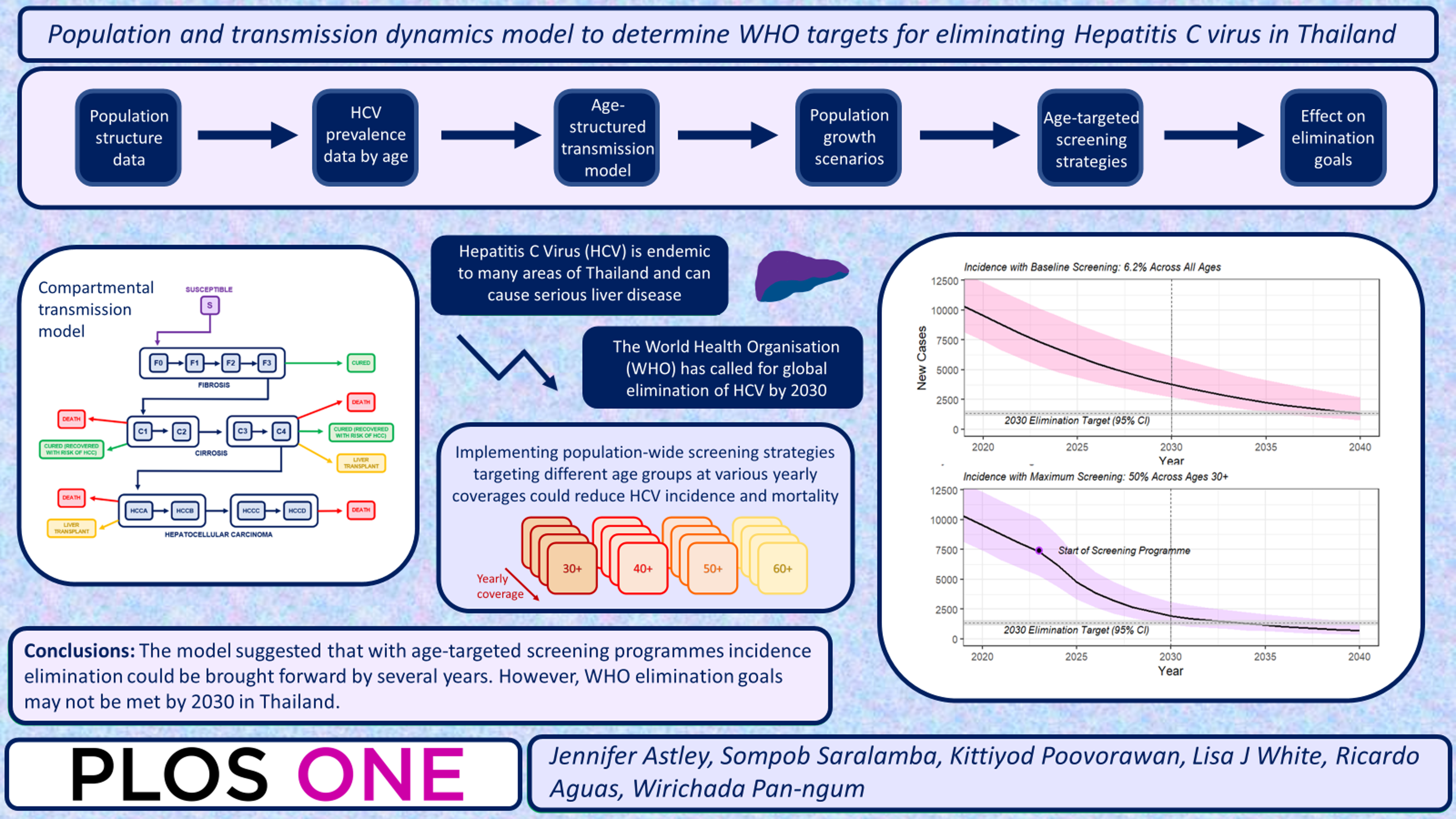

Supplement: S1 Graphical abstract — (TIFF) [file pone.0309313.s012.tiff]
