# Supplementary material for: Population and transmission dynamics model to determine WHO targets for eliminating Hepatitis C virus in Thailand
Source: PLoS One. 2024 Oct 16;19(10):e0309313. doi: 10.1371/journal.pone.0309313 (PMC11482681; doi:10.1371/journal.pone.0309313)
Supplement: S2 File — Complete list of all Ordinary Differential Equations specifying the transmission model. Complete list of all compartments in the transmission model. Complete list of all parameters in model with description, parameter name (in model code), value and source. Further detail on methods used in all aspects of the work done to produce these results. (DOCX) [file pone.0309313.s010.docx]

Supplementary file B

System of Ordinary Differential Equations describing HCV transmission and aging in Thailand

Individuals alive **AL**:

$$\boldsymbol{AL}=\boldsymbol{S}+\boldsymbol{F}\boldsymbol{0}+\boldsymbol{F}\boldsymbol{1}+\boldsymbol{F}\boldsymbol{2}+\boldsymbol{F}\boldsymbol{3}+\boldsymbol{C}\boldsymbol{1}+\boldsymbol{C}\boldsymbol{2+C3+C}\boldsymbol{4+HCCA+HCCB+HCCC+HCCD+ F}\boldsymbol{0}\boldsymbol{cure+F}\boldsymbol{1}\boldsymbol{cure+F}\boldsymbol{2}\boldsymbol{cure+F}\boldsymbol{3}\boldsymbol{cure+C}\boldsymbol{1}\boldsymbol{cure+C}\boldsymbol{2}\boldsymbol{cure+C}\boldsymbol{3}\boldsymbol{cure}+\boldsymbol{C}\boldsymbol{4}\boldsymbol{cure}$$

Total population:

$$population= \sum\boldsymbol{AL}$$

Total infected individuals ***I***:

$$\boldsymbol{I=F}\boldsymbol{0+F}\boldsymbol{1+F2+F}\boldsymbol{3+C}\boldsymbol{1+C}\boldsymbol{2+C}\boldsymbol{3+C}\boldsymbol{4}$$

Transmission matrix (beta) 𝔹:

$$\mathbb{B}=0.01*\mathbb{T}$$

Force of infection $\mathbb{L}$:

$$\mathbb{L}=\frac{\mathbb{B}*\boldsymbol{I}}{population}$$

Prevalence (%):

$$prev= 100* \frac{\boldsymbol{I}}{population}$$

Ordinary Differential Equations:

$$\frac{d\boldsymbol{S}}{dt}= \boldsymbol{\sigma}- \boldsymbol{\lambda S}-\boldsymbol{\mu S}+\mathbb{A}\boldsymbol{S}$$

$$\frac{d\boldsymbol{F}\boldsymbol{0}}{dt}= -f0f1 \boldsymbol{F}\boldsymbol{0}+\mathbb{L}\boldsymbol{S}-\boldsymbol{scr}*pF0scr*sens*cureF0*\boldsymbol{F}\boldsymbol{0- \mu F}\boldsymbol{0+}\mathbb{A}\boldsymbol{F}\boldsymbol{0}$$

$$\frac{d\boldsymbol{F}\boldsymbol{1}}{dt}=f0f1 \boldsymbol{F}\boldsymbol{0}-f1f2 \boldsymbol{F}\boldsymbol{1}-\boldsymbol{scr}*pF1scr*sens*cureF1*\boldsymbol{F}\boldsymbol{1- \mu F}\boldsymbol{1+}\mathbb{A}\boldsymbol{F}\boldsymbol{1}$$

$$\frac{d\boldsymbol{F}\boldsymbol{2}}{dt}=f1f2 \boldsymbol{F}\boldsymbol{2}-f2f3 \boldsymbol{F}\boldsymbol{2}-\boldsymbol{scr}*pF2scr*sens*cureF2-\boldsymbol{\mu F}\boldsymbol{2+}\mathbb{A}\boldsymbol{F}\boldsymbol{2}$$

$$\frac{d\boldsymbol{F}\boldsymbol{3}}{dt}=f2f3 \boldsymbol{F}\boldsymbol{3}\boldsymbol{-}f3c1 \boldsymbol{F}\boldsymbol{3}-\boldsymbol{scr}*pF3scr*sens*cureF3*\boldsymbol{F}\boldsymbol{3}-\boldsymbol{\mu F}\boldsymbol{3+}\mathbb{A}\boldsymbol{F}\boldsymbol{3}$$

$$\frac{d\boldsymbol{C}\boldsymbol{1}}{dt}=f3c1\boldsymbol{F}\boldsymbol{3}-dthc1 \boldsymbol{C}\boldsymbol{1}-c1c2\boldsymbol{C}\boldsymbol{1}-\boldsymbol{scr}*pC1scr*sens*cureC1*\boldsymbol{C}\boldsymbol{1-(}c1bA+c1bB+c1bC+c1bD\boldsymbol{)C}\boldsymbol{1}-\boldsymbol{\mu C}\boldsymbol{1+}\mathbb{A}\boldsymbol{C}\boldsymbol{1}$$

$$\frac{d\boldsymbol{C}\boldsymbol{2}}{dt}=c1c2 \boldsymbol{C}\boldsymbol{1}-dthc2 \boldsymbol{C}\boldsymbol{2}-c2c3 \boldsymbol{C}\boldsymbol{2}-\boldsymbol{scr}*pC2scr*sens*cureC2*\boldsymbol{C}\boldsymbol{2}-\left( c2bA+c2bB+c2bC+c2bD \right)\boldsymbol{C}\boldsymbol{2} -\boldsymbol{\mu C}\boldsymbol{2+}\mathbb{A}\boldsymbol{C}\boldsymbol{2}$$

$$\frac{d\boldsymbol{C}\boldsymbol{3}}{dt}=c2c3 \boldsymbol{C}\boldsymbol{2}-dthc3 \boldsymbol{C}\boldsymbol{3}-c3c4 \boldsymbol{C}\boldsymbol{3}-\boldsymbol{src}*pC3scr*sens*cureC3*\boldsymbol{C}\boldsymbol{3}-c3bD \boldsymbol{C}\boldsymbol{3}-\boldsymbol{\mu C}\boldsymbol{3+}\mathbb{A}\boldsymbol{C}\boldsymbol{3}$$

$$\frac{d\boldsymbol{C}\boldsymbol{4}}{dt}=c3c4 \boldsymbol{C}\boldsymbol{3}-dthc4 \boldsymbol{C}\boldsymbol{4}-\boldsymbol{scr}*pC4scr*sens*cureC4 \boldsymbol{C}\boldsymbol{4}-c4bD \boldsymbol{C}\boldsymbol{4}-tranc4 \boldsymbol{C}\boldsymbol{4} -\boldsymbol{\mu C}\boldsymbol{4+}\mathbb{A}\boldsymbol{C}\boldsymbol{4}$$

$$\frac{d\boldsymbol{HCCA}}{dt}=c1bA\left( \boldsymbol{C}\boldsymbol{1+C}\boldsymbol{1}\boldsymbol{cure} \right)+c2bA\left( \boldsymbol{C}\boldsymbol{2+C}\boldsymbol{2}\boldsymbol{cure} \right)-dthbA \boldsymbol{HCCA}-tranbA \boldsymbol{HCCA}-\boldsymbol{\mu HCCA+}\mathbb{A}\boldsymbol{HCCA}$$

$$\frac{d\boldsymbol{HCCB}}{dt}=c1bB\left( \boldsymbol{C}\boldsymbol{1+C}\boldsymbol{1}\boldsymbol{cure} \right)+c2bB\left( \boldsymbol{C}\boldsymbol{2+C}\boldsymbol{2}\boldsymbol{cure} \right)-dthbB \boldsymbol{HCCB}-tranbB \boldsymbol{HCCB}-\boldsymbol{\mu HCCB+}\mathbb{A}\boldsymbol{HCCB}$$

$$\frac{d\boldsymbol{HCCC}}{dt}=c1bC\left( \boldsymbol{C}\boldsymbol{1+C}\boldsymbol{1}\boldsymbol{cure} \right)+c2bC\left( \boldsymbol{C}\boldsymbol{2+C}\boldsymbol{2}\boldsymbol{cure} \right)-dthbC \boldsymbol{HCCC}-\boldsymbol{\mu HCCC+}\mathbb{A}\boldsymbol{HCCC}$$

$$\frac{d\boldsymbol{HCCD}}{dt}=c1bD\left( \boldsymbol{C}\boldsymbol{1+C}\boldsymbol{1}\boldsymbol{cure} \right)+c2bD\left( \boldsymbol{C}\boldsymbol{2+C}\boldsymbol{2}\boldsymbol{cure} \right)+c3bD\left( \boldsymbol{C}\boldsymbol{3+C}\boldsymbol{3}\boldsymbol{cure} \right)+c4bD\left( \boldsymbol{C}\boldsymbol{4+C}\boldsymbol{4}\boldsymbol{cure} \right)-dthbD \boldsymbol{HCCD}-\boldsymbol{\mu HCCD+}\mathbb{A}\boldsymbol{HCCD}$$

$$\frac{d\boldsymbol{F}\boldsymbol{0}\boldsymbol{cure}}{dt}=\boldsymbol{scr}*pF0scr*sens*cureF0*\boldsymbol{F}\boldsymbol{0}-\boldsymbol{\mu F}\boldsymbol{0}\boldsymbol{cure+}\mathbb{A}\boldsymbol{F}\boldsymbol{0}\boldsymbol{cure}$$

$$\frac{d\boldsymbol{F}\boldsymbol{1}\boldsymbol{cure}}{dt}=\boldsymbol{scr}*pF1scr*sens*cureF1*\boldsymbol{F}\boldsymbol{1}-\boldsymbol{\mu F}\boldsymbol{1}\boldsymbol{cure+}\mathbb{A}\boldsymbol{F}\boldsymbol{1}\boldsymbol{cure}$$

$$\frac{d\boldsymbol{F}\boldsymbol{2}\boldsymbol{cure}}{dt}=\boldsymbol{scr}*pF2scr*sens*cureF2*\boldsymbol{F}\boldsymbol{2}-\boldsymbol{\mu F}\boldsymbol{2}\boldsymbol{cure+}\mathbb{A}\boldsymbol{F}\boldsymbol{2}\boldsymbol{cure}$$

$$\frac{d\boldsymbol{F}\boldsymbol{3}\boldsymbol{cure}}{dt}=\boldsymbol{scr}*pF3scr*sens*cureF3*\boldsymbol{F}\boldsymbol{3}-\boldsymbol{\mu F}\boldsymbol{3}\boldsymbol{cure+}\mathbb{A}\boldsymbol{F}\boldsymbol{3}\boldsymbol{cure}$$

$$\frac{d\boldsymbol{C}\boldsymbol{1}\boldsymbol{cure}}{dt}=\boldsymbol{scr}*pC1scr*sens*cureC1*\boldsymbol{C}\boldsymbol{1}-\left( c1bA+c1bB+c1bC+c1bD \right) \boldsymbol{C}\boldsymbol{1}\boldsymbol{cure}-\boldsymbol{\mu C}\boldsymbol{1}\boldsymbol{cure+}\mathbb{A}\boldsymbol{C}\boldsymbol{1}\boldsymbol{cure}$$

$$\frac{d\boldsymbol{C}\boldsymbol{2}\boldsymbol{cure}}{dt}=\boldsymbol{scr}*pC2scr*sens*cureC2*\boldsymbol{C}\boldsymbol{2}-\left( c2bA+c2bB+c2bC+c2bD \right) \boldsymbol{C}\boldsymbol{2}\boldsymbol{cure}-\boldsymbol{\mu C}\boldsymbol{2}\boldsymbol{cure+}\mathbb{A}\boldsymbol{C}\boldsymbol{2}\boldsymbol{cure}$$

$$\frac{d\boldsymbol{C}\boldsymbol{3}\boldsymbol{cure}}{dt}=\boldsymbol{scr}*pC3scr*sens*cureC3*\boldsymbol{C}\boldsymbol{3}-c3bD \boldsymbol{C}\boldsymbol{3}\boldsymbol{cure}-\boldsymbol{\mu C}\boldsymbol{3}\boldsymbol{cure+}\mathbb{A}\boldsymbol{C}\boldsymbol{3}\boldsymbol{cure}$$

$$\frac{d\boldsymbol{C}\boldsymbol{4}\boldsymbol{cure}}{dt}=\boldsymbol{scr}*pC4scr*sens*cureC4*\boldsymbol{C}\boldsymbol{4}-c4bD \boldsymbol{C}\boldsymbol{4}\boldsymbol{cure}-\boldsymbol{\mu C}\boldsymbol{4}\boldsymbol{cure+}\mathbb{A}\boldsymbol{C}\boldsymbol{4}\boldsymbol{cure}$$

$$\frac{d\boldsymbol{D}}{dt}=dthc1 \boldsymbol{C}\boldsymbol{1}+dthc2 \boldsymbol{C}\boldsymbol{2}+dthc3 \boldsymbol{C}\boldsymbol{3}+dthc4 \boldsymbol{C}\boldsymbol{4}+dthbA \boldsymbol{HCCA}+dthbB \boldsymbol{HCCB}+dthbC \boldsymbol{HCCC}+dthbD \boldsymbol{HCCD}$$

$$\frac{d\boldsymbol{CInc}}{dt}=\boldsymbol{\lambda S}$$

Key:

Vectors of length 21: ***μ, σ, F0*** etc.

Matrices of dimension 21x21: 𝔸, 𝕃, 𝔹 etc.

Scalar parameters: *f0f1, sens, cureC1* etc

Table S1: Transmission compartments

| Compartment | Description |
| --- | --- |
| S | Susceptible to Hepatitis C Virus infection (assumed to be whole population except those already infected) |
| F0 | Fibrosis stage 0 |
| F1 | Fibrosis stage 1 |
| F2 | Fibrosis stage 2 |
| F3 | Fibrosis stage 3 |
| C1 | Cirrhosis stage 1: Child-Pugh class A |
| C2 | Cirrhosis stage 2: Child-Pugh class A |
| C3 | Cirrhosis stage 3: Child-Pugh class B |
| C4 | Cirrhosis stage 4: Child-Pugh class C |
| HCCA | Hepatocellular carcinoma stage A |
| HCCB | Hepatocellular carcinoma stage B |
| HCCC | Hepatocellular carcinoma stage C |
| HCCD | Hepatocellular carcinoma stage D |
| D | Cumulative HCV deaths |
| dthC14 | Cumulative deaths from cirrhosis stages 1-4 |
| dthHCC | Cumulative deaths from HCC stages A-D |
| transliv | Cumulative liver transplants |

Table S2: Parameter list

| **Data** | **Parameter** | **Value** | **Description** | **Source** |
| --- | --- | --- | --- | --- |
| Model parameter | start_year | 2004 | Starting year of model simulation | - |
| Transition rates (years^-1^) | f0f1 | 0.117 | Transition F0 to F1 | (Poovorawan et al., 2016) |
|  | f1f2 | 0.085 | Transition F1 to F2 |  |
|  | f2f3 | 0.12 | Transition F2 to F3 |  |
|  | f3c1 | 0.116 | Transition F3 to C1 |  |
|  | c1c2 | 0.044 | Transition C1 to C2 |  |
|  | c2c3 | 0.044 | Transition C2 to C3 |  |
|  | c3c4 | 0.076 | Transition C3 to C4 |  |
|  | c1bA | 0.0068 | Transition C1 to HCCA |  |
|  | c1bB | 0.0099 | Transition C1 to HCCB |  |
|  | c1bC | 0.0029 | Transition C1 to HCCC |  |
|  | c1bD | 0.0068 | Transition C1 to HCCD |  |
|  | c2bA | 0.0068 | Transition C2 to HCCA |  |
|  | c2bB | 0.0099 | Transition C2 to HCCB |  |
|  | c2bC | 0.0029 | Transition C2 to HCCC |  |
|  | c2bD | 0.0068 | Transition C2 to HCCD |  |
|  | dthc1 | 0.01 | Death from C1 |  |
|  | dthc2 | 0.01 | Death from C2 |  |
|  | dthc3 | 0.2 | Death from C3 |  |
|  | dthc4 | 0.57 | Death from C4 |  |
|  | dthbA | 1/(36/12) | Death from HCCA |  |
|  | dthbB | 1/(16/12) | Death from HCCB |  |
|  | dthbC | 1/(6/12) | Death from HCCC |  |
|  | dthbD | 1/(3/12) | Death from HCCD |  |
|  | dthtrn | 1/(240/12) | Death from liver transplant |  |
|  | tranc4 | 0.0015 | Liver transplant rate from C4 |  |
|  | tranbA | 0.0015 | Liver transplant rate from HCCA |  |
|  | tranbB | 0.0015 | Liver transplant rate from HCCB |  |
| Current treatment parameters | trt_start | 15 | Start year of current DAA treatment (15 = 2019) | (Poovorawan et al., 2016) |
|  | sens | 0.985 | Sensitivity of screening process (simplified: combined tests) |  |

Table S2 (continued)

| Screening probabilities | pF0scr | 1 | Probability of being screened in F0 if in screening programme | Assumed by model |
| --- | --- | --- | --- | --- |
|  | pF1scr | 1 | Probability of being screened in F1 if in screening programme |  |
|  | pF2scr | 1 | Probability of being screened in F2 if in screening programme |  |
|  | pF3scr | 1 | Probability of being screened in F3 if in screening programme |  |
|  | pC1scr | 1 | Probability of being screened in C1 if in screening programme |  |
|  | pC2scr | 1 | Probability of being screened in C2 if in screening programme |  |
|  | pC3scr | 1 | Probability of being screened in C3 if in screening programme |  |
|  | pC4scr | 1 | Probability of being screened in C4 if in screening programme |  |
| Treatment efficacies | std_cureF0 | 0.7 | Efficacy of old treatment of F0 (PEGs) | (Poovorawan et al., 2016) |
|  | std_cureF1 | 0.7 | Efficacy of old treatment of F1 (PEGs) |  |
|  | std_cureF2 | 0.7 | Efficacy of old treatment of F2 (PEGs) |  |
|  | std_cureF3 | 0.7 | Efficacy of old treatment of F3 (PEGs) |  |
|  | std_cureC1 | 0.7 | Efficacy of old treatment of C1 (PEGs) |  |
|  | new_cureF0 | 0.985 | Efficacy of current treatment of F0 (DAAs) |  |
|  | new_cureF1 | 0.985 | Efficacy of current treatment of F1 (DAAs) |  |
|  | new_cureF2 | 0.985 | Efficacy of current treatment of F2 (DAAs) |  |
|  | new_cureF3 | 0.985 | Efficacy of current treatment of F3 (DAAs) |  |
|  | new_cureC1 | 0.985 | Efficacy of current treatment of C1 (DAAs) |  |
|  | new_cureC2 | 0.985 | Efficacy of current treatment of C2 (DAAs) |  |
|  | new_cureC3 | 0.985 | Efficacy of current treatment of C3 (DAAs) |  |
|  | new_cureC4 | 0.985 | Efficacy of current treatment of C4 (DAAs) |  |

Table S2 (continued)

| Screening strategy parameters | scr_start | 19 | Start year of screening programme (19=2023) | Under test |
| --- | --- | --- | --- | --- |
|  | scr_dur | 5 | Duration of screening programme (years) | Under test |
|  | cov_mean | 0.03 | Baseline coverage mean (assumed normal distribution) | Estimated by model |
|  | cov_sd | 0.02 | Baseline coverage standard deviation (assumed normal distribution) | Estimated by model |
|  | cov_lower | cov_mean + 1.96*cov_sd | Lower bound for baseline coverage (95% CI) | Calculated |
|  | cov_upper | cov_mean - 1.96*cov_sd | Upper bound for baseline coverage (95% CI) | Calculated |
|  | coverage1 | 0.5 | Yearly screening coverage tested | Under test |
|  | coverage2 | 0.9 | Yearly screening coverage tested | Under test |

Supplementary Methods

***Manipulation of Data***

*Mortality Rate*

Raw data on mortality rates were provided in units of *total deaths by single age, both sexes combined (thousands).* The single age values were combined into 5-year age groups and multiplied by 1000 to obtain number of deaths per year for each age group and year from 2004 – 2021. Each value was then divided by the total population in that age group for that year to obtain a mortality rate with unit *deaths per person per year*. The *approx.* interpolation function was used to estimate a mortality function for each individual age group to give a value for natural deaths at each time step of the model. These were combined into a vector of length 21 to multiply by each compartment in order to model natural death from that compartment.

*Birth Rate*

Raw birth rate data were given in births per 1000 for each year, with data for 2004-2021 and projections for 2022 – 2040. The values were divided by 1000 to obtain a birth rate with unit *births per person per year* which was used in the model. The *approx.* interpolation function was used to estimate a birth rate value for each time step in the model.

*Sexual Contact*

The small diagonal matrix from the work by Chanthavilay et al., 2016 relating to sexual mixing by age was extrapolated to 21 age groups, with 0.6 on the diagonal and 0.4 elsewhere representing the probability of sexual contact between the same or different age groups. This diagonal matrix was then multiplied by the mean number of annual sexual partners by age group, from which an average of male and female had been taken. Finally, this matrix was multiplied by 0.025 in order to produce transmission parameter (beta) values reflecting those of previous models (Poovorawan et al., 2016).

*Prevalence Data*

Data from a study estimating Thai prevalence by age group in 2004 and 2014 based on a collection of serum samples were used to calibrate the model (Wasitthankasem et al., 2016). The sample size for this study was 5964 which was used to calculate the 95% confidence intervals around each data point, using equation (1), where z is the z score (1.96 for 95% CI), p is the population proportion (the prevalence in this instance), n is the sample size (5964) and N is the population size (number of individuals in the given age group and year).

1. $CI^{'}=p\pm z \times\sqrt{\frac{p(1-p)}{n'}\times\frac{N-n'}{N-1}}$

*Population Data*

Population data by age group were provided in units of total individuals by M/F, age group and year from 2004 to 2021. The Male and Female values were added to obtain an absolute population count for each age group in each year. These values were divided by the total population in the same year to calculate the proportion of the total population held in each age group in any given year. The same source also provided a projection from 2022-2040 of the same values. These data points were used to validate the model and ensure that population structure was preserved.

*Initial Conditions*

The population by age group according to United Nations data was preserved in the initial conditions, with the start year of the model simulation being 2004. Prevalence data by age group from the work of Wasitthankasem et al., 2016 were used to estimate the number of infections in each age group in 2004. Infections were assumed to be evenly distributed across the fibrosis and cirrhosis stages according to three larger age groupings: infections in ages 0-30 were all F0 (early stage fibrosis only); infections in ages 30-59 were all F0-F3 (no cirrhosis or HCC), and prevalence in ages 95+ was zero since individuals likely wouldn’t live to that age with untreated infection.
