# Supplementary material for: Population and transmission dynamics model to determine WHO targets for eliminating Hepatitis C virus in Thailand
Source: PLoS One. 2024 Oct 16;19(10):e0309313. doi: 10.1371/journal.pone.0309313 (PMC11482681; doi:10.1371/journal.pone.0309313)
Supplement: S3 File — Further detail on results obtained from this model that are additional to main results. (DOCX) [file pone.0309313.s011.docx]

Supplementary file C – Supplementary Results


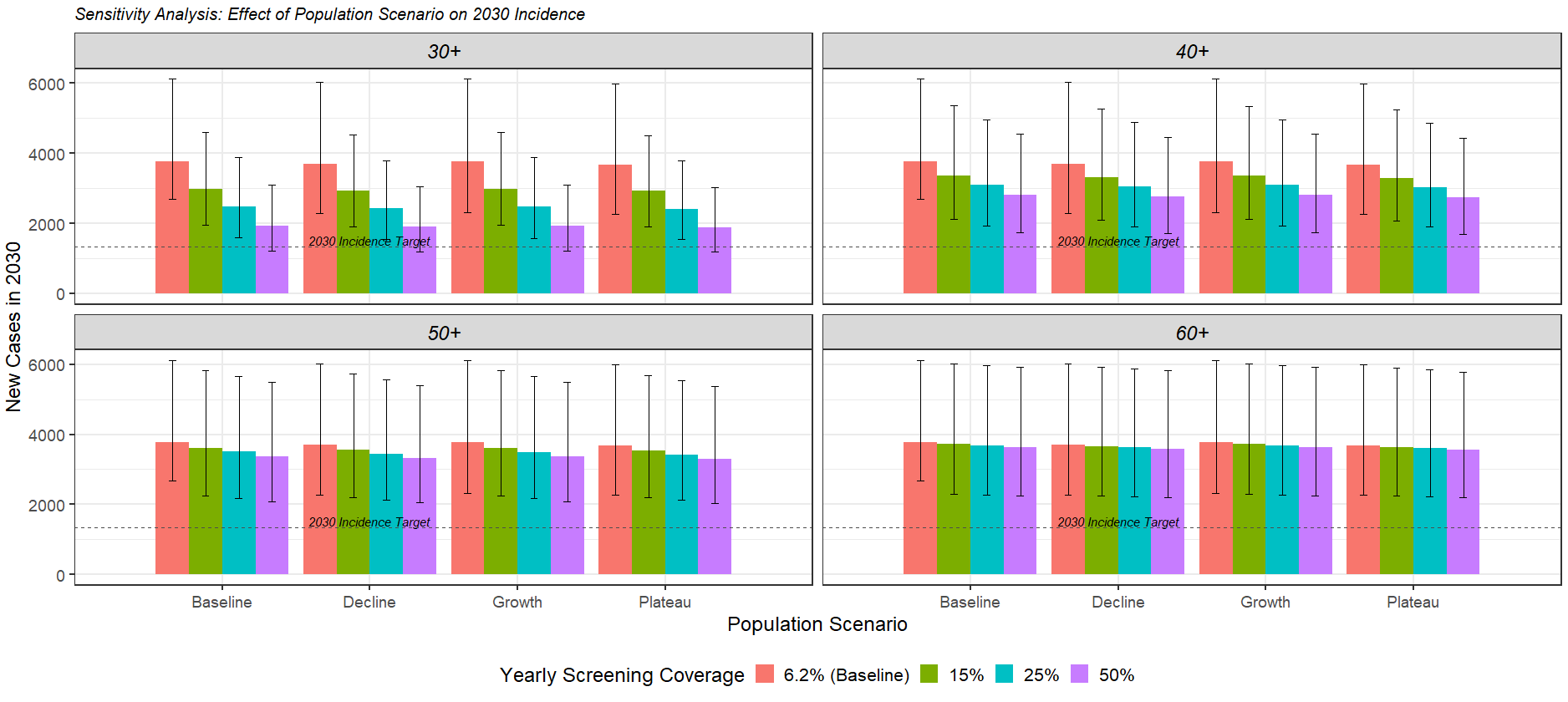


Figure S3: Bar chart showing the difference in 2030 incidence for each population scenario, screening coverage and targeted age group


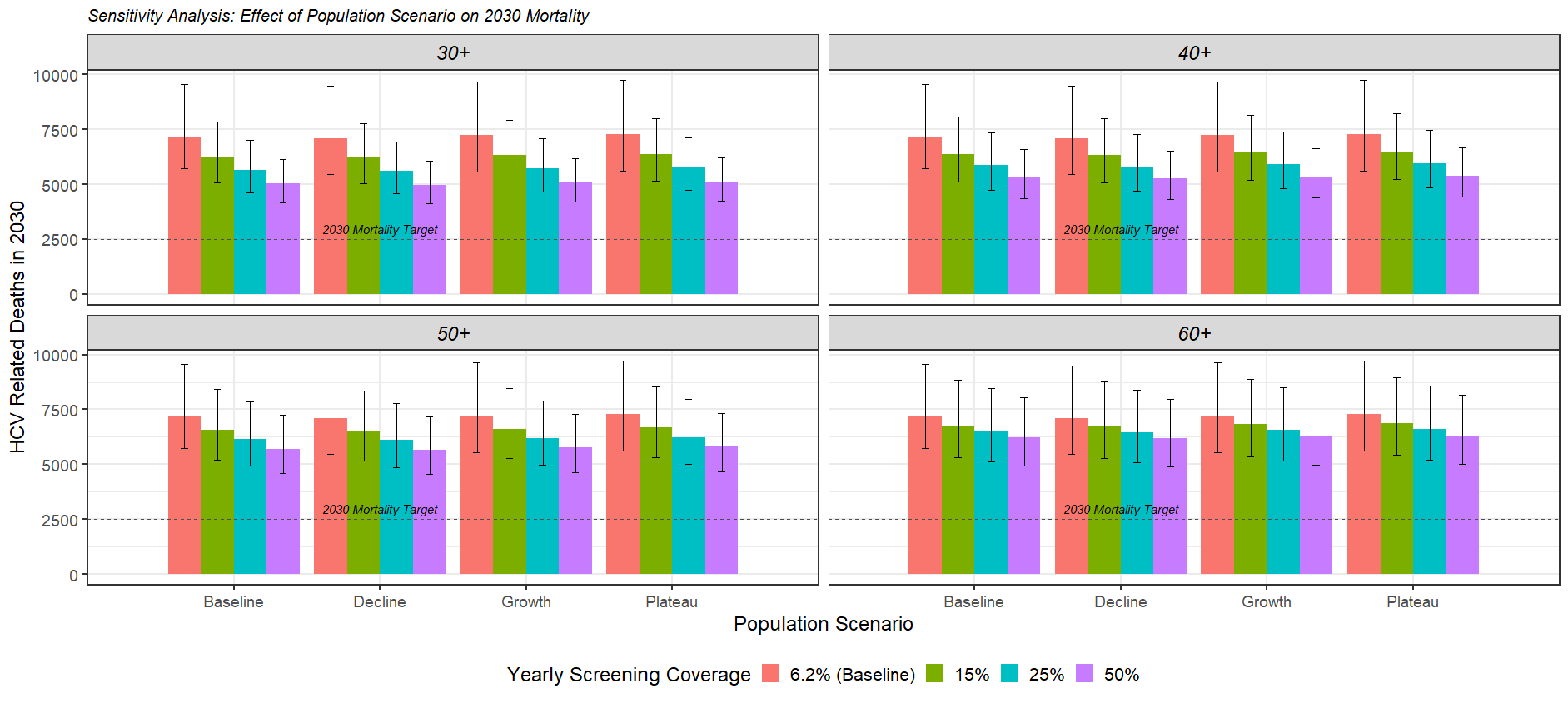


Figure S4: Bar chart showing the difference in 2030 mortality for each population scenario, screening coverage and targeted age group


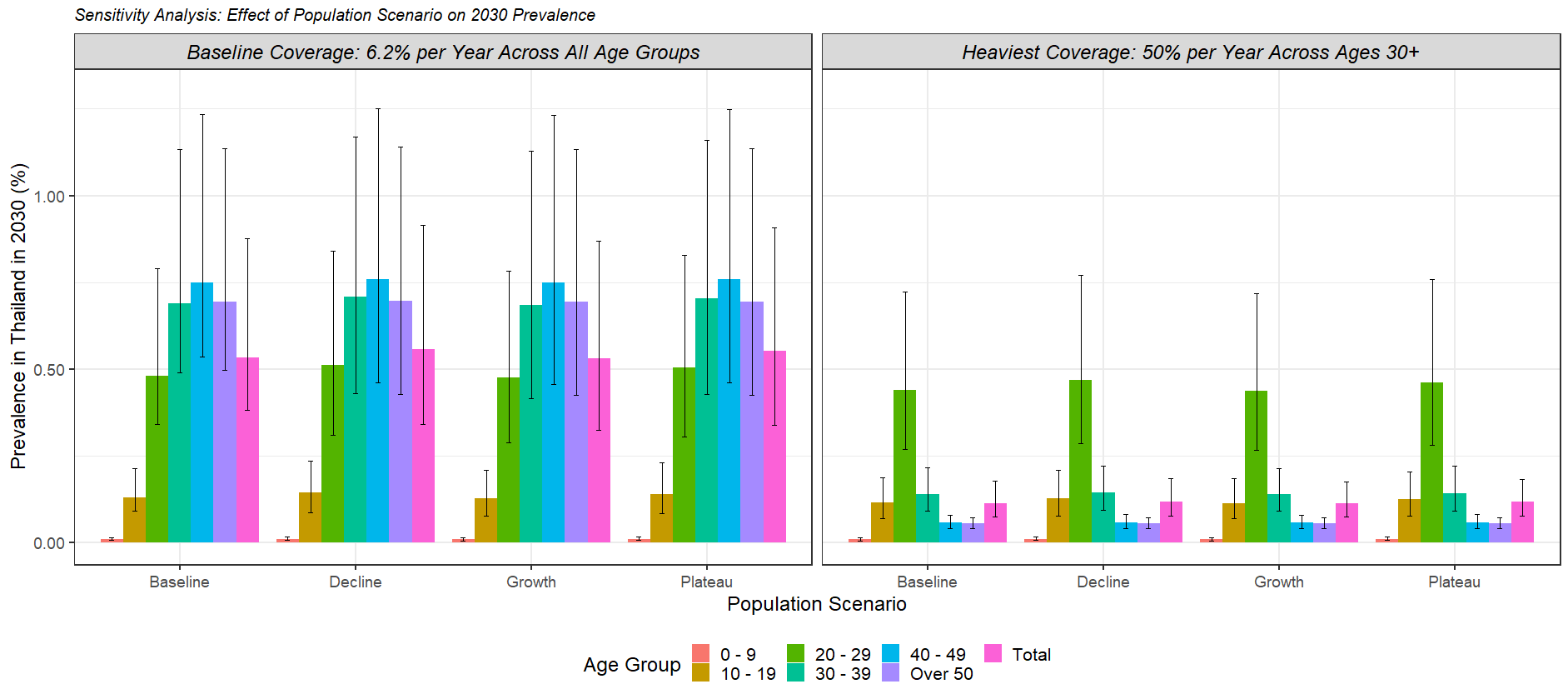


Figure S5: Bar chart showing the difference in 2030 prevalence across all age groups for baseline and most extreme screening strategies between population scenarios


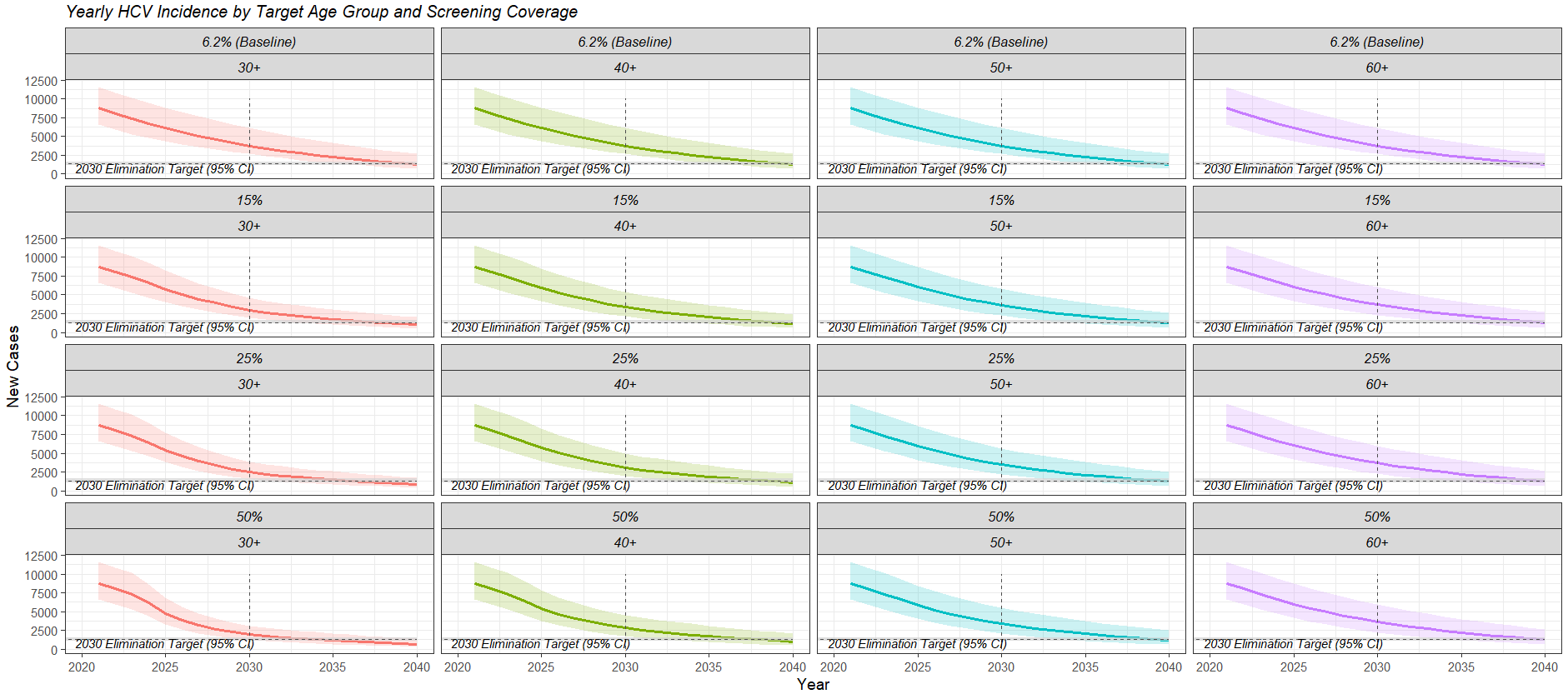


Figure S6: Model output of incidence compared to WHO 2030 goals for all screening coverages and target age groups at baseline population scenario


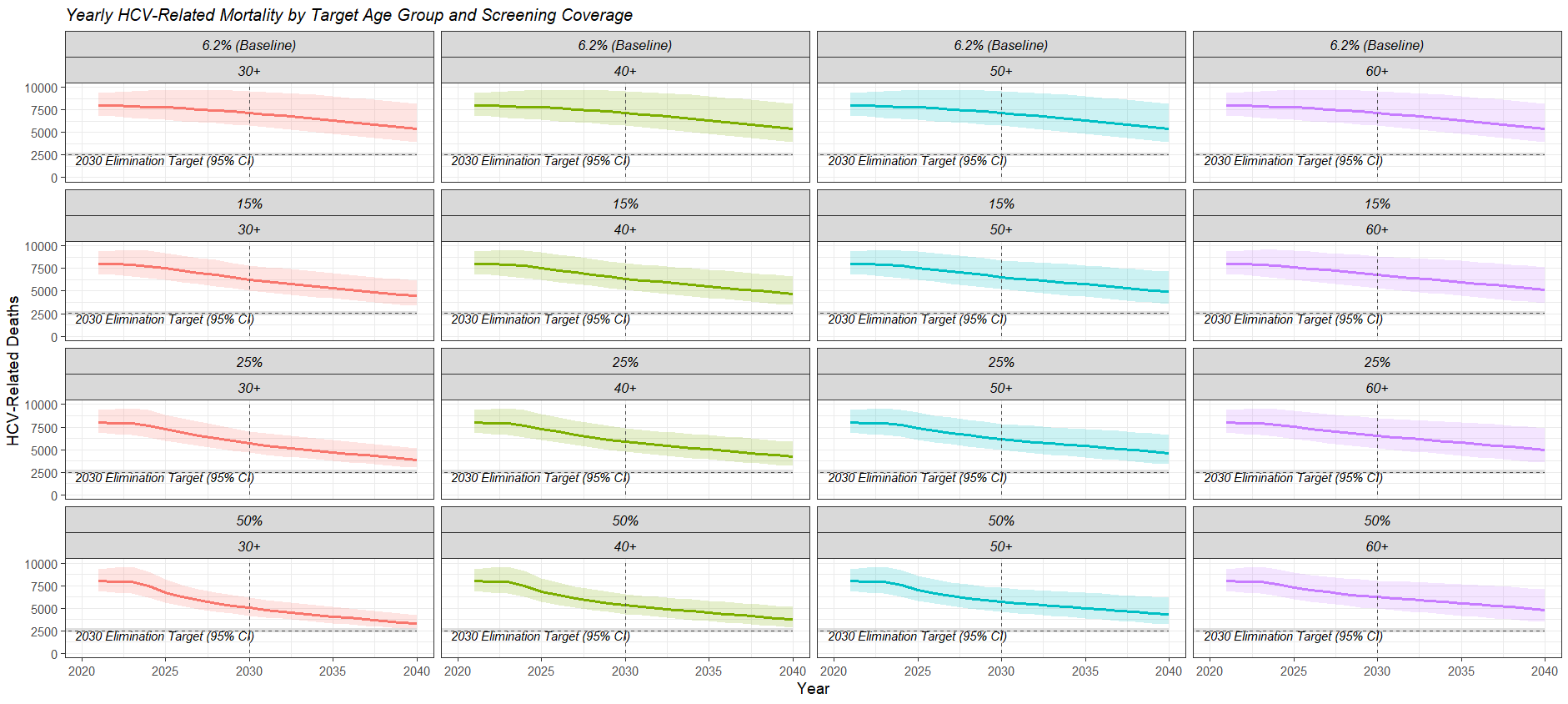


Figure S7: Model output of mortality compared to WHO 2030 goals for all screening coverages and target age groups at baseline population scenario


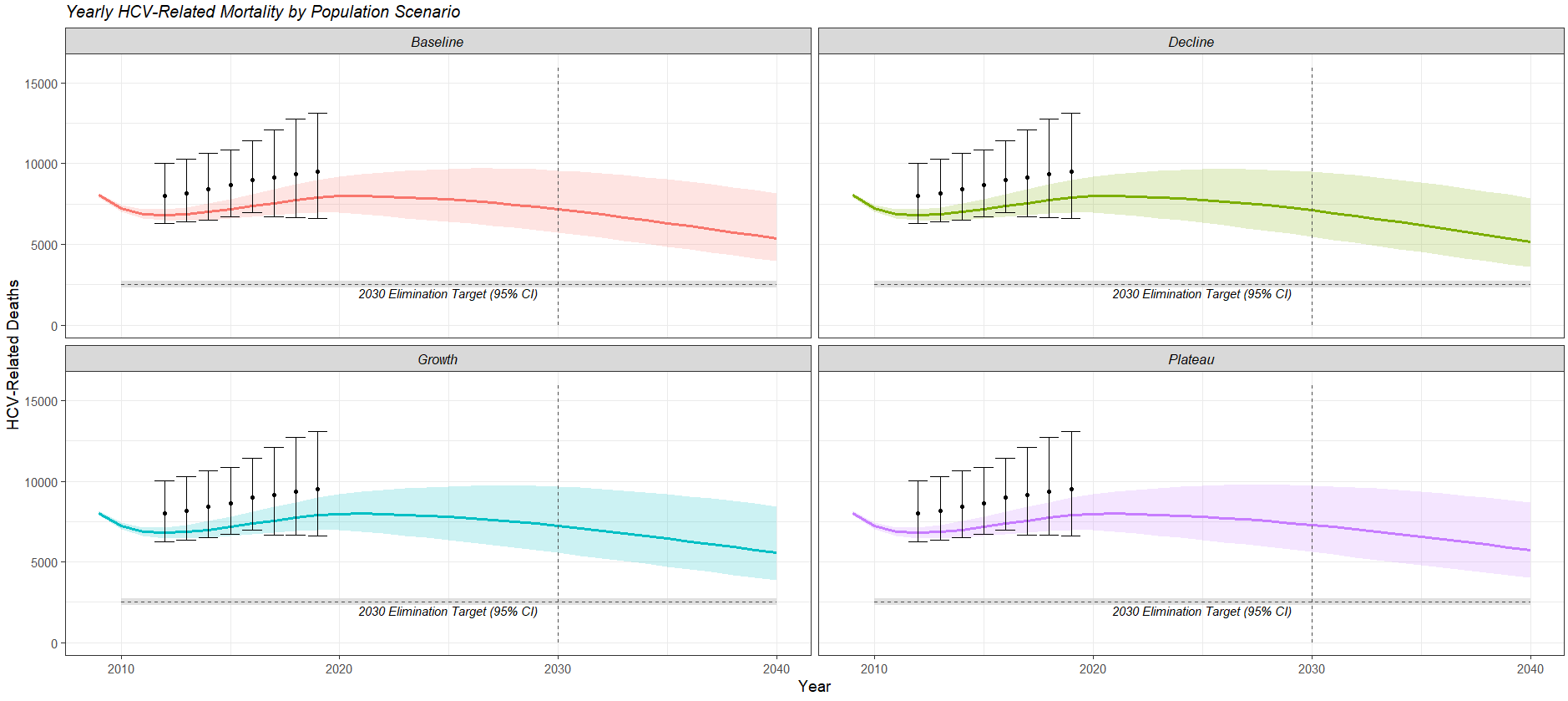


Figure S8: Model output of mortality at baseline screening coverage for all four populations scenarios compared with 2030 WHO goals and model output from Coalition for Global Hepatitis Elimination and World Health Organisation, 2019
